# The PERK/PKR-eIF2α Pathway Negatively Regulates Porcine Hemagglutinating Encephalomyelitis Virus Replication by Attenuating Global Protein Translation and Facilitating Stress Granule Formation

**DOI:** 10.1128/JVI.01695-21

**Published:** 2022-01-12

**Authors:** Junchao Shi, Zi Li, Rongyi Xu, Jing Zhang, Qianyu Zhou, Rui Gao, Huijun Lu, Yungang Lan, Kui Zhao, Hongbin He, Feng Gao, Wenqi He

**Affiliations:** a Key Laboratory of Zoonosis Research, Ministry of Education, College of Veterinary Medicine, Jilin University, Changchun, China; b Key Laboratory of Zoonosis Research, Ministry of Education, Institute of Zoonosis, Jilin University, Changchun, China; c Key Laboratory of Animal Resistant Biology of Shandong, Ruminant Disease Research Center, College of Life Sciences, Shandong Normal University, Jinan, China; Loyola University Chicago

**Keywords:** coronavirus, porcine hemagglutinating encephalomyelitis virus, endoplasmic reticulum stress, eIF2α, translation attenuation, stress granule

## Abstract

The replication of coronaviruses, including severe acute respiratory syndrome coronavirus (SARS-CoV), Middle East respiratory syndrome coronavirus (MERS-CoV), and the recently emerged severe acute respiratory syndrome coronavirus 2 (SARS-CoV-2), is closely associated with the endoplasmic reticulum (ER) of infected cells. The unfolded protein response (UPR), which is mediated by ER stress (ERS), is a typical outcome in coronavirus-infected cells and is closely associated with the characteristics of coronaviruses. However, the interaction between virus-induced ERS and coronavirus replication is poorly understood. Here, we demonstrate that infection with the betacoronavirus porcine hemagglutinating encephalomyelitis virus (PHEV) induced ERS and triggered all three branches of the UPR signaling pathway both *in vitro* and *in vivo*. In addition, ERS suppressed PHEV replication in mouse neuro-2a (N2a) cells primarily by activating the protein kinase R-like ER kinase (PERK)–eukaryotic initiation factor 2α (eIF2α) axis of the UPR. Moreover, another eIF2α phosphorylation kinase, interferon (IFN)-induced double-stranded RNA-dependent protein kinase (PKR), was also activated and acted cooperatively with PERK to decrease PHEV replication. Furthermore, we demonstrate that the PERK/PKR-eIF2α pathways negatively regulated PHEV replication by attenuating global protein translation. Phosphorylated eIF2α also promoted the formation of stress granules (SGs), which in turn repressed PHEV replication. In summary, our study presents a vital aspect of the host innate response to invading pathogens and reveals attractive host targets (e.g., PERK, PKR, and eIF2α) for antiviral drugs.

**IMPORTANCE** Coronavirus diseases are caused by different coronaviruses of importance in humans and animals, and specific treatments are extremely limited. ERS, which can activate the UPR to modulate viral replication and the host innate response, is a frequent occurrence in coronavirus-infected cells. PHEV, a neurotropic betacoronavirus, causes nerve cell damage, which accounts for the high mortality rates in suckling piglets. However, it remains incompletely understood whether the highly developed ER in nerve cells plays an antiviral role in ERS and how ERS regulates viral proliferation. In this study, we found that PHEV infection induced ERS and activated the UPR both *in vitro* and *in vivo* and that the activated PERK/PKR-eIF2α axis inhibited PHEV replication through attenuating global protein translation and promoting SG formation. A better understanding of coronavirus-induced ERS and UPR activation may reveal the pathogenic mechanism of coronavirus and facilitate the development of new treatment strategies for these diseases.

## INTRODUCTION

The harmful effects of coronaviruses on humans are widely known. In particular, the harmful effects of coronavirus disease 2019 (COVID-19), which is caused by severe acute respiratory syndrome coronavirus 2 (SARS-CoV-2) and has been the cause of great panic (1, 2), and those of outbreaks of severe acute respiratory syndrome coronavirus (SARS-CoV) and Middle East respiratory syndrome coronavirus (MERS-CoV) infection in previous years are well known (3, 4). Similarly, numerous emerging and reemerging pathogenic coronaviruses, such as transmissible gastroenteritis virus (TGEV), porcine epidemic diarrhea virus (PEDV), porcine deltacoronavirus (PDCoV), and porcine hemagglutinating encephalomyelitis virus (PHEV), are extremely harmful to pig farming worldwide, revealing an urgent need to understand the pathogenesis and develop prevention and control strategies (5–7).

PHEV is a type of porcine contagious neurotropic coronavirus belonging to the genus *Betacoronaviru*s within the family *Coronaviridae* of the order *Nidovirales* (8, 9). Similar to other coronaviruses of the same genus, PHEV can also cause respiratory symptoms in pigs (10, 11). In contrast, PHEV is typically neurotropic and is the only known neurotropic coronavirus capable of infecting pigs currently (9). The mortality rate in suckling piglets within 3 weeks of age can reach 100%, and infected piglets present obvious neurological symptoms. According to epidemiological investigations, PHEV infection is endemic and highly prevalent worldwide (12), and with the increasing number of reported cases, its harm to the pig industry is also receiving increasing attention. Since the clinical characteristics and neuropathological changes in mice and rats infected with PHEV are similar to those in piglets, mice and rats have been widely used to study the pathogenesis of PHEV (13–16). PHEV can cause neurodegeneration and even the loss and death of cerebral cortical and hippocampal neurons in mice after intranasal inoculation (17). Further studies have revealed that PHEV infection leads to neuronal dysplasia, unstable formation of dendritic spines, and irregular expansion and disconnection of neurites (18). However, during the process of neuronal injury induced by PHEV, the antiviral response and function of the cells themselves are not clear.

Endoplasmic reticulum (ER) stress (ERS) is an adaptive cell response that enables cells to activate the unfolded protein response (UPR) to restore homeostasis when the misfolded and unfolded proteins aggregate in the ER lumen (19). The UPR reaction is mainly mediated by three kinds of transmembrane proteins responsible for signal transduction: inositol-requiring enzyme 1 (IRE1), protein kinase R-like ER kinase (PERK), and activation of transcription factor 6 (ATF6) (19, 20). When the UPR is triggered, ER molecular chaperones (such as GRP78 and calnexin) separate from the above-mentioned three transmembrane proteins. Next, IRE1, PERK, and ATF6 are activated, which in turn transmit signals to the cytoplasm and nucleus through different downstream signaling molecules, resulting in reduced protein synthesis and enhanced misfolded protein degradation (19–22). However, the continuous activation of the UPR signal can also induce an endogenous apoptotic response, which eventually results in cell death (19). In addition, ERS plays a crucial part in the pathogenesis of various coronavirus diseases through interactions with immune regulation, autophagy, and apoptotic pathways (23–27).

Stress granules (SGs) are dynamic, nonmembranous cytosolic RNA granules generated within the cytoplasm of eukaryotic cells to respond to various environmental stresses such as virus infection and ERS (28). SGs are made up of translationally silenced mRNAs, 40S ribosome subunits, and RNA-binding proteins. Ras-GTPase-activating protein-binding protein 1 (G3BP1), T cell intracellular antigen 1 (TIA1), and poly(A)-binding protein (PABP) are three fundamental components of SGs and are proposed to be markers for SGs (28). Although the functional role of SGs in viral infection remains controversial, accumulating evidence indicates that SGs participate in the host cell antiviral response to restrict viral propagation (29–32).

PHEV is a neurotropic coronavirus that is transferred from the peripheral nervous system (PNS) to the central nervous system (CNS), and the virus can successfully evade innate immunity during the course of infection (13, 16, 33, 34). The regeneration ability of nerve cells is very weak, so we speculate that when nerve cells are under virus infection, their antiviral response ability may also play a prominent role. Whether the defense systems of nerve cells play a role in PHEV infection remains unknown. Considering the significance of the ER in coronavirus propagation and the abundance of ER (Nissl body) in nerve cells, we investigated the ability of PHEV to trigger ERS in nerve cells and how this phenomenon affected viral replication. We found that PHEV triggered ERS and activated all three branches of UPR signaling *in vitro* and *in vivo*. Furthermore, we demonstrated that ERS suppressed PHEV replication, mainly by activating the PERK/interferon (IFN)-induced double-stranded RNA-dependent protein kinase (PKR)–eukaryotic initiation factor 2α (eIF2α) pathway to reduce global protein synthesis and facilitate the generation of SGs. Our findings not only reveal the molecular mechanism underlying PHEV pathogenicity but also provide new drug targets with scientific significance and practical value.

## RESULTS

### PHEV infection alters the ER structure.

**FIG 1:**
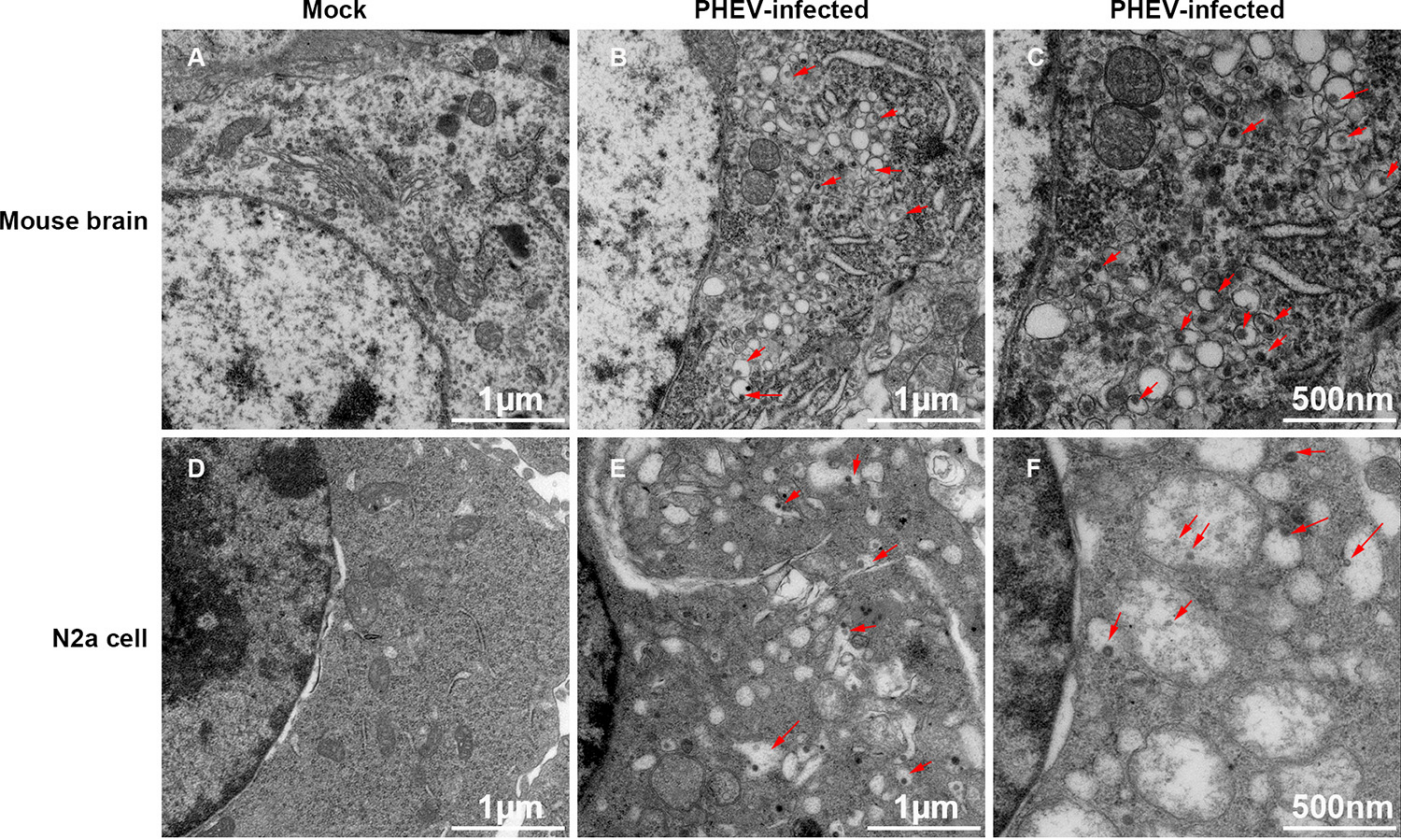
TEM analysis of PHEV-infected mice and cells. (A to C) Mice were mock or PHEV infected by intranasal inoculation for 5 days. When obvious neurological symptoms were present, the brains were collected for TEM analysis. (A) Mock-infected mouse brain. (B and C) PHEV-infected mouse brain. (D to F) N2a cells were mock infected or infected with PHEV for 24 h, and the cells were fixed for TEM analysis. (D) Mock-infected N2a cells. (E and F) PHEV-infected N2a cells. The red arrows indicate the virions in the vesicles of the ER. Three mice from each group were used for TEM analysis, and representative electron micrographs are shown.

To investigate whether PHEV infection triggered an ERS response, transmission electron microscopy (TEM) was conducted to determine whether PHEV infection altered the ER structure. Compared with the control group (Fig. 1A and D), the appearance of dilated and hyperswollen membranous lamellae of the ER was evident throughout the majority of PHEV-infected neuro-2a (N2a) cells (Fig. 1B and C) and mouse brains (Fig. 1E and F). In the diluted cisternae of the ER, numerous scattered virus-like particles were present (Fig. 1C and F). These observations confirmed that PHEV infection caused excessive alterations of the ER structure both *in vitro* and *in vivo*.

### PHEV infection induces ERS.

**FIG 2:**
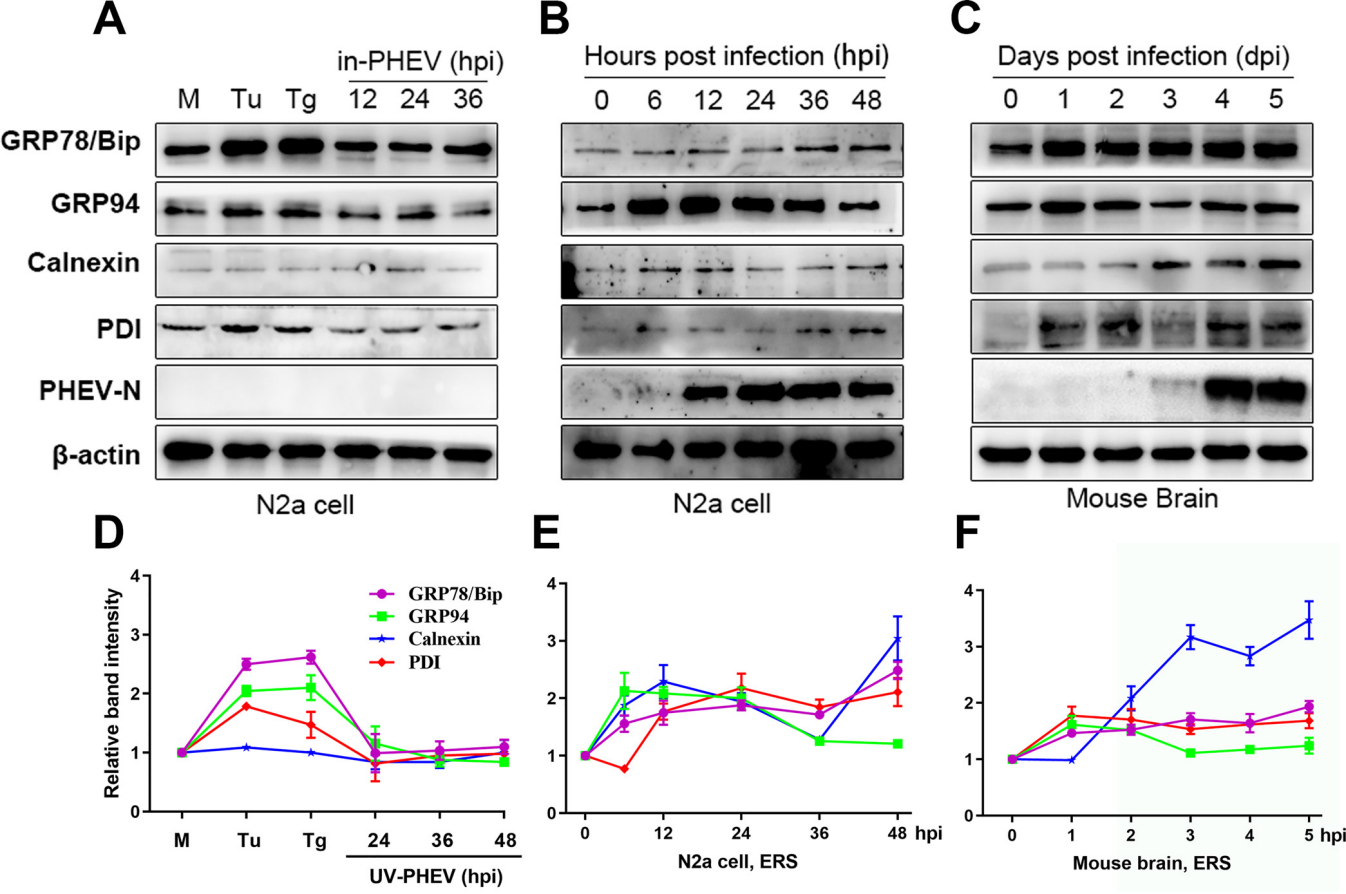
PHEV infection induces ERS both *in vitro* and *in vivo*. (A) Untreated N2a cells (M), cells treated with 2 μg/mL Tu or 1 μM Tg for 12 h as positive controls, and cells infected with heat-inactivated PHEV (in-PHEV) for 12, 24, and 36 h as negative controls. (B and C) PHEV-infected N2a cells and mouse brain samples were harvested at different time points. The ERS marker protein expression levels were determined by Western blotting. (D to F) Quantification of the ERS marker proteins during PHEV infection. The abundances of Bip/GRP78, GRP94, calnexin, and PDI are expressed as fold changes compared with the mock-infected control after normalization to β-actin. As an indication of infection, PHEV N protein was used. Results are representative of data from three independent experiments, and data in panels D to F are expressed as the means ± SD.

We next investigated the expression levels of the major ERS markers (GRP78/Bip, GRP94, calnexin, and PDI) following PHEV infection in N2a cells and mouse brains. Cells treated with the ERS inducers tunicamycin (Tu) and thapsigargin (Tg) were used as positive controls. Compared with an untreated control, treatment with Tu or Tg resulted in elevated expression levels of the chaperones GRP78/Bip, GRP94, and PDI in N2a cells (Fig. 2A and D). Interestingly, as previously shown, calnexin displayed unchanged expression profiles (Fig. 2A and D) (35). In the experimental group, PHEV-infected N2a cells produced higher expression levels of all ERS marker proteins than did mock-infected cells (Fig. 2B and E). To further verify the induction of ERS *in vivo*, we monitored the expression level of ERS markers as described above in mouse brains following PHEV infection. Mice are widely used as an animal model to study the pathogenesis of PHEV since the clinical symptoms and neuropathological changes in mice infected with PHEV are similar to those in piglets (18, 33, 36). An upregulation of these ERS markers was also noted in PHEV-infected mice (Fig. 2C and F). Heat-inactivated PHEV (in-PHEV) failed to induce the upregulation of ERS marker proteins in N2a cells compared with actively replicating PHEV (Fig. 2A and D), suggesting that PHEV-induced ERS was dependent on infectious virus. Overall, these findings showed that PHEV infection caused ERS both *in vitro* and *in vivo*.

### PHEV infection triggers all three channels of the UPR signaling pathway *in vitro*.

**FIG 3:**
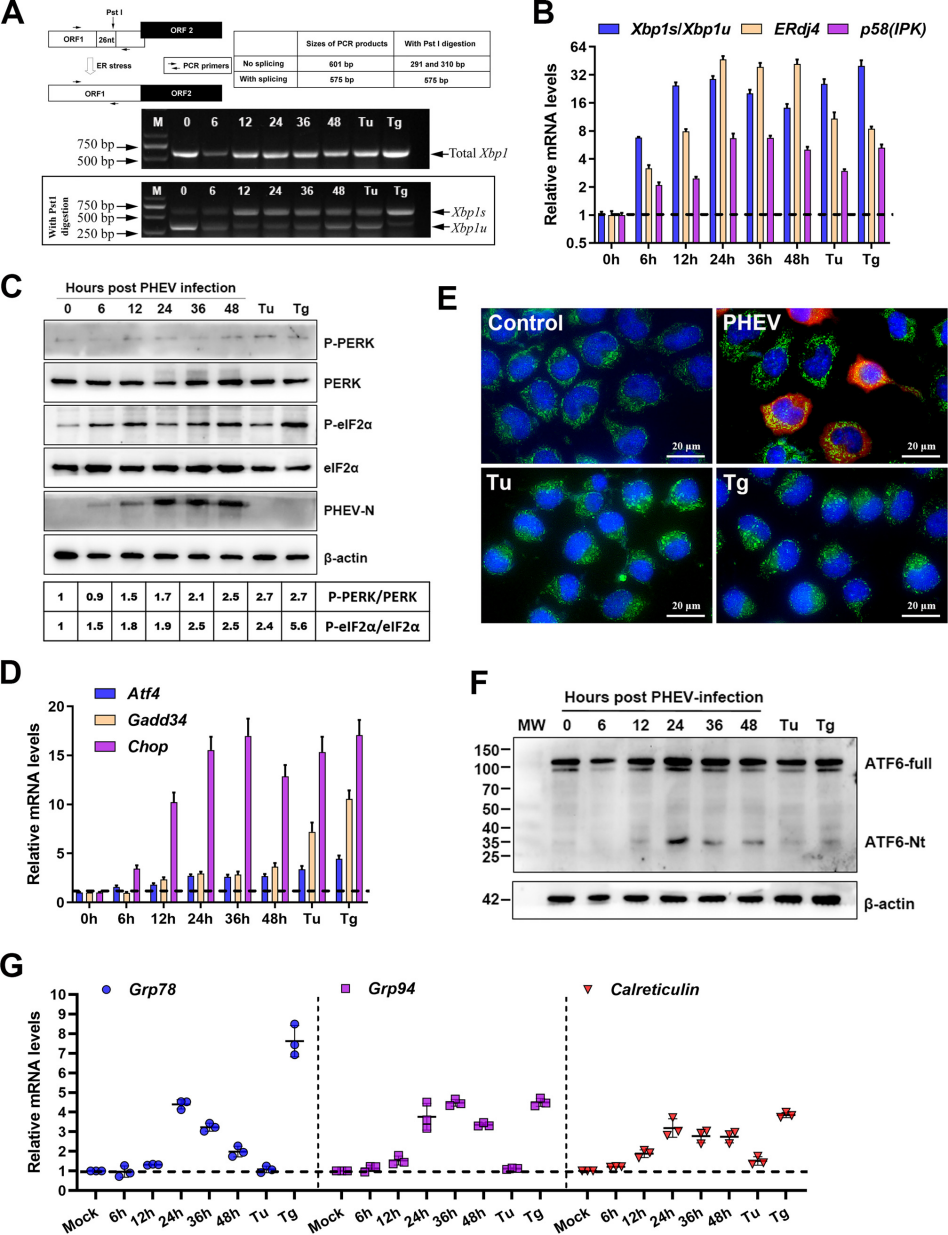
*In vitro*, PHEV infection stimulates all three branches of UPR signaling. (A) PHEV infection causes *Xbp1* mRNA splicing in N2a cells. The sizes of spliced *Xbp1* (*Xbp1s*) and unspliced *Xbp1* (*Xbp1u*) products with or without PstI restriction enzyme cleavage are shown in the schematic diagram. Tu (2 μg/mL)- and Tg (1 μM)-treated and mock-infected or PHEV-infected N2a cells were collected at the indicated times for RT-PCR and PstI digestion analysis, and the products were analyzed by electrophoresis on a 2% agarose gel. nt, nucleotides; ORF1, open reading frame 1. (B) qRT-PCR analysis of IRE1 downstream genes [*Xbp1*, *ERdj4*, and *p^58^*^(^*^IPK^*^)^]. Data are normalized by GAPDH and presented as fold changes in expression relative to mock (dashed line). (C) Western blot analysis of PERK, p-PERK, eIF2α, and p-eIF2α in PHEV-infected N2a cells. Mock-infected or PHEV-infected N2a cells were harvested at 6, 12, 24, 36, or 48 h for Western blotting. Tu (2 μg/mL)- and Tg (1 μM)-treated cells were used as positive controls. As an indicator of infection, anti-PHEV N antibody was employed, and β-actin was used as the loading control. The band intensities for p-PERK and p-eIF2α were normalized to those for total PERK and total eIF2α, respectively. Below the blots, the fold changes in phosphorylation are shown, with phosphorylation assigned a value of 1 at 0 hpi. (D) qRT-PCR analysis of PERK pathway downstream genes (*Atf4*, *Chop*, and *Gadd34*). Data are normalized as described above for panel B. (E) Representative immunofluorescence images of N2a cells mock infected, infected with PHEV for 24 h, treated with Tu (2 μg/mL) for 2 h or Tg (1 μM) for 2 h, and incubated with anti-ATF6 (green) and anti-PHEV N (red) antibodies. Nuclei were counterstained with DAPI (blue). (F) Western blot analysis of cleaved ATF6 (ATF6-Nt). MW, molecular weight (in thousands). (G) qRT-PCR analysis of *Grp78*, *Grp94*, and *Calreticulin* mRNAs. Data are normalized as described above for panel B. Results are representative of data from three independent experiments, and data in panels B, D, and G are expressed as the means ± SD.

To restore ER homeostasis, three ER sensors (IRE1, PERK, and ATF6) of the UPR signaling pathway are triggered. Thus, following PHEV infection, we initially assessed the status of the three ER sensors of the UPR signaling pathway. Upon activation by oligomerization and autophosphorylation of IRE1, the endoribonuclease activity of IRE1 is activated and selectively cleaves a 26-bp intron from unspliced X-box-binding protein 1 (*Xbp1u*) mRNA, resulting in a spliced and frameshifted transcript that encodes an active spliced X-box-binding protein 1 (XBP1s) transcription factor, the target genes of which boost the folding capacities of the ER (37). The splicing of *Xbp1u* mRNA causes the deletion of the PstI restriction site located within the intron (27). To monitor the IRE1 branch of the UPR, reverse transcription-PCR (RT-PCR) and restriction enzyme cleavage assays were performed using specific primers flanking the splice site and PstI enzyme, respectively (Fig. 3A). After PHEV infection, *Xbp1s* gradually increased, peaked at 12 h, and then decreased from approximately 24-fold to 16-fold in the later phase of infection (Fig. 3A and B). *ERdj4* and *p^58^*^(^*^IPK^*^)^, two transcriptionally induced downstream genes of *Xbp1s* (38), were also increased substantially in N2a cells infected with PHEV (Fig. 3B). These results showed that PHEV infection activated the IRE1-XBP1 axis *in vitro*.

PERK is a key molecule among the three sensors of the UPR. Under ERS, the activated PERK phosphorylates eIF2α at serine 51, which blocks the conversion of inactive GDP-bound eIF2α to the active GTP-bound form, thereby limiting translation initiation and leading to global translation attenuation (25). However, in this circumstance, ATF4 translation is enhanced, resulting in the induction of its target genes *Chop* and *Gadd34* (24, 25). To monitor PERK activation, the phosphorylation statuses of PERK and eIF2α were analyzed by Western blotting utilizing antibodies specific for PERK, phosphorylated PERK (p-PERK), eIF2α, and p-eIF2α during PHEV infection. The levels of phosphorylated PERK were marginally elevated with time, peaking at 48 h postinfection (hpi). Interestingly, phosphorylated eIF2α reached a maximum level at 36 h and was then stable, possibly due to GADD34-mediated dephosphorylation (Fig. 3C). As expected, the eIF2α downstream mRNA (*Atf4*, *Chop*, and *Gadd34*) levels were also elevated (Fig. 3D). These results suggested that the PERK-ATF4-CHOP axis was also activated in PHEV-infected N2a cells.

When activated, ATF6 translocates to the Golgi apparatus, where the proteases cleave 90-kDa full-length ATF6 (p90ATF6) (ATF6-full), and the 50-kDa active variant of ATF6 consisting of the N terminus (p50ATF6) (ATF6-Nt) is released (37). After cleavage, p50ATF6 translocates into the nucleus to switch ER chaperone genes (such as *Grp78*/*Bip*, *Grp94*, and *Calreticulin*) (25). To explore whether PHEV infection activated the ER sensor of ATF6, immunofluorescence (IF) and Western blot assays were conducted to detect ATF6 nuclear translocation and cleavage, respectively. However, we were unable to detect clear nuclear translocation in both PHEV-infected and positive-control (Tu- and Tg-treated) cells (Fig. 3E); a possible reason is that this antibody did not recognize mouse ATF6-Nt efficiently in immunofluorescence assays. Moreover, bands of the trimmed version of ATF6 (ATF6-Nt) also could not be detected obviously (Fig. 3F). Alternatively, we monitored the induction of *Grp78*, *Grp94*, and *Calreticulin*, which were known to be induced by ATF6-Nt. Compared with Tg, Tu treatment did not induce significant induction of all three of these transcripts in N2a cells (Fig. 3G). An increase in *Grp78* mRNA was observed in Tg-treated and, to a lesser extent, in PHEV-infected cells. Interestingly, the mRNA level of *Grp78* decreased from 24 to 48 hpi (Fig. 3G). Nevertheless, increased mRNA levels of *Grp94* and *Calreticulin* were detected in PHEV-infected cells and compatible with those in Tg-treated positive cells (Fig. 3G), indicating that ATF6 was activated following PHEV infection. In conclusion, the three UPR signaling pathways were all activated to different extents after PHEV infection *in vitro*.

### PHEV infection triggers all three channels of the UPR signaling pathway *in vivo*.

**FIG 4:**
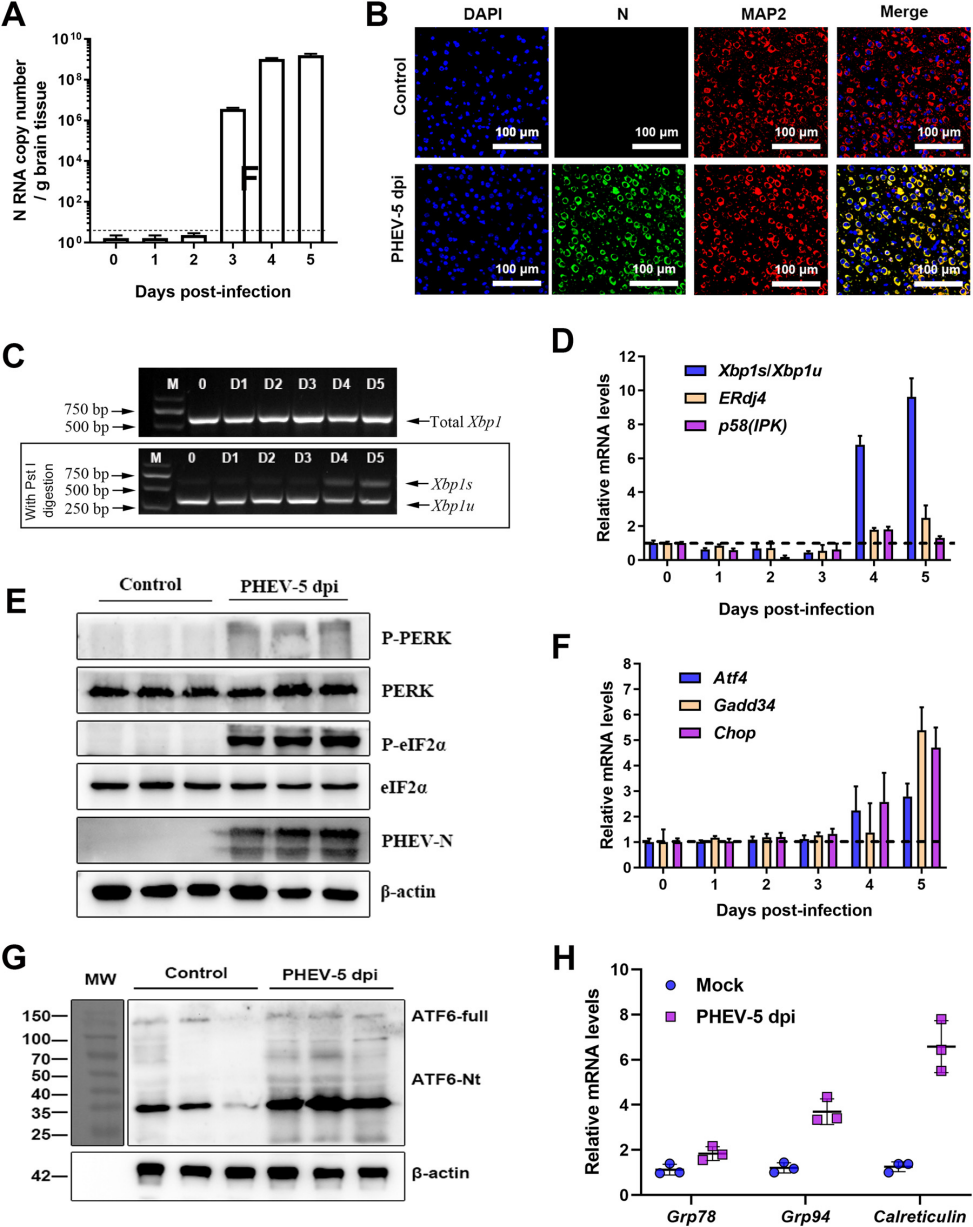
PHEV infection activates all three branches of the UPR in the mouse brain. Mice were intranasally inoculated with PHEV or mock infected with the same dose of DMEM. All mice were euthanized, and the brains were harvested at 5 days postinfection (dpi). (A) qRT-PCR was used to assess virus replication in the brain. (B) Representative immunofluorescence photographs of viral antigens in mock- and PHEV-infected brain tissues. MAP2, microtubule-associated protein 2. Nuclei are counterstained with DAPI (blue). (C) *Xbp1* mRNA splicing analysis in mock- and PHEV-infected mouse brains. (D) Ratios of *Xbp1s*/*Xbp1u* and mRNA expression levels of IRE1 downstream genes [*ERdj4* and *p^58^*^(^*^IPK^*^)^] in brain samples. Data are normalized as described in the legend of Fig. 3B. (E) Western blot analysis of p-PERK, PERK, p-eIF2α, and eIF2α in brain samples from control and PHEV-infected mice. (F) qRT-PCR analysis of PERK downstream genes (*Atf4*, *Chop*, and *Gadd34*) in mouse brain samples. Data are normalized as described in the legend of Fig. 3B. (G) Western blot analysis of cleaved ATF6 (ATF6-Nt) in brain samples. (H) qRT-PCR analysis of *Grp78*, *Grp94*, and *Calreticulin* mRNAs. Data are normalized described in the legend of Fig. 3B. Data in panels A, D, F, and H are representative of results from three independent experiments and are shown as the means ± SD.

To further investigate whether PHEV infection activated all three sensors of the UPR *in vivo*, the three UPR molecules were analyzed in PHEV-infected mouse brains. Viral loads in mouse brains were measured by quantitative RT-PCR (qRT-PCR) (Fig. 4A) and immunofluorescence staining with anti-PHEV N protein antibody to confirm PHEV infection (Fig. 4B). *Xbp1* splicing and induction of its target genes could be detected only after 4 days postinfection (dpi) (Fig. 4C and D), suggesting that IRE1 was activated in the later stage of PHEV infection *in vivo*. In PHEV-infected brain samples (5 dpi), phosphorylated PERK and phosphorylated eIF2α were also substantially increased relative to the control samples (Fig. 4E), suggesting the activation of the PERK signaling pathway. Consistent with these findings, the mRNA levels of *Atf4*, *Chop*, and *Gadd34* were also increased after PHEV infection at 5 dpi (Fig. 4F). *In vivo*, specific bands of ATF6-Nt were not detected in mock- and PHEV-infected mouse brains (Fig. 4G); the results were consistent with those *in vitro*. Even so, a significant increase in *Grp78*, *Grp94*, and *Calreticulin* transcription was observed in PHEV-infected brain samples (Fig. 4H), indicating that the ATF6 pathway was activated after PHEV infection. Collectively, PHEV infection triggered all three branches of UPR signaling *in vivo*.

### ERS is detrimental to PHEV replication in N2a cells.

**FIG 5:**
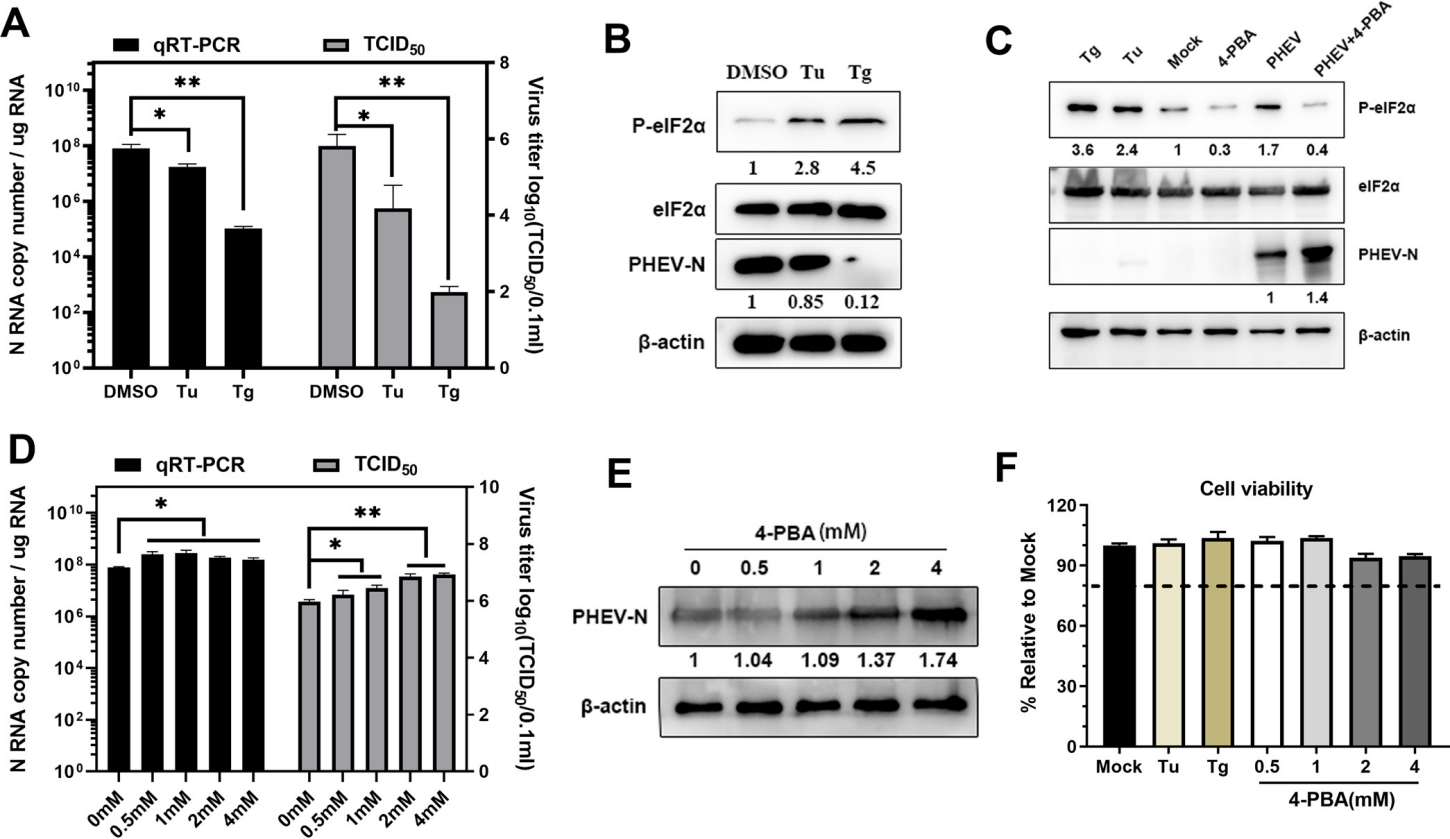
ERS inhibits PHEV replication in N2a cells. Tu, Tg, 4-PBA, or DMSO was used to pretreat N2a cells 2 h prior to infection and kept at the same concentration after infection. (A and D) PHEV N RNA copy numbers and virus titers. (B, C, and E) p-eIF2α, eIF2α, and PHEV N expression was tested using Western blotting. Below the immunoblots are quantifications of PHEV N and p-eIF2α, normalized by β-actin and eIF2α, respectively, and expressed relative to mock. (F) Cells were treated with the indicated concentrations of Tu, Tg, and 4-PBA, and cell viability was measured using a CCK-8 assay. In panels A, D, and F, the error bars represent the SD from three independent experiments. *, *P* ≤ 0.05; **, *P* ≤ 0.01.

Coronavirus replication is structurally and functionally linked to the ER, and increasing evidence has shown that the coronavirus-induced UPR mediated by ERS regulates viral replication (27, 39–41). To uncover the effects of ERS on PHEV replication, Tu and Tg, two commonly used chemical ERS inducers, were used. Treatment with Tu (2 μg/mL) or Tg (1 μM) in N2a cells substantially inhibited PHEV infection, and Tg had a more significant anti-PHEV effect than Tu (Fig. 5A). PHEV replication inhibition by Tu or Tg was further verified by Western blotting using an anti-PHEV N protein antibody, and the expression levels of eIF2α and p-eIF2α were also detected (Fig. 5B). No significant cytotoxicity was discovered by evaluating cell viability using a cell counting kit 8 (CCK-8) test, indicating that the suppression of viral replication by Tg or Tu was not due to cell cytotoxicity (Fig. 5F).

To better understand the function of ERS in PHEV replication, we pretreated N2a cells with 4-phenylbutyric acid (4-PBA), a chemical chaperone that has been reported to reduce ERS by restricting eIF2α phosphorylation (42, 43). In PHEV-infected N2a cells, 4-PBA pretreatment lowered eIF2α phosphorylation (Fig. 5C), indicating that 4-PBA alleviated PHEV-induced ERS. Treatment with 4-PBA boosted dose-dependent PHEV replication as evidenced by viral RNA levels, virus titers, and PHEV N protein levels (Fig. 5C to E). The ability of 4-PBA treatment to improve PHEV replication suggested that alleviating ERS by preventing eIF2α phosphorylation aided PHEV propagation. Since CCK-8 assays showed that 4-PBA had no influence on cell viability (Fig. 5F), the increased PHEV replication following 4-PBA treatment was not due to an improvement in cell viability. Collectively, these findings demonstrated that extra UPR activation was harmful to viral replication.

### PHEV replication inhibition is not linked with the triggered IRE1 and ATF6 pathways.

**FIG 6:**
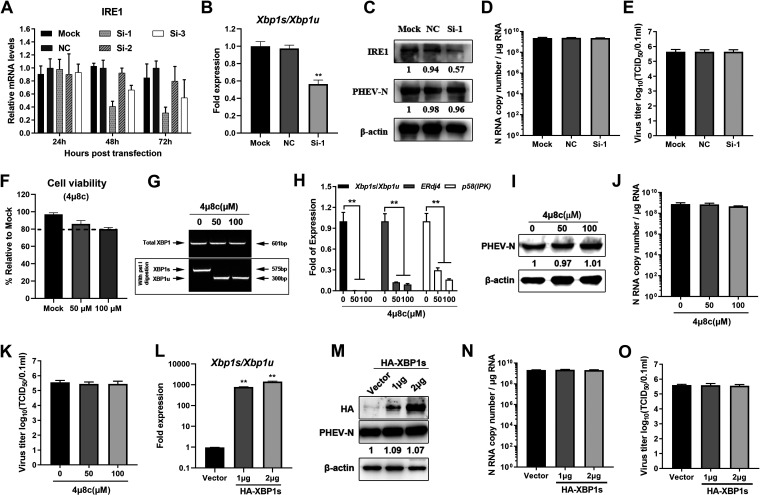
The IRE1 pathway is not responsible for the inhibition of PHEV replication. (A) N2a cells were transfected with siRNA specific for IRE1 for 24 h, 48 h, and 72 h, and qRT-PCR was used to determine the best interference efficiency. (B to E) N2a cells were transfected with NC and siRNA 1 targeting IRE1 for 48 h and then infected with PHEV. *Xbp1* mRNA splicing assays (B), Western blotting (C), qRT-PCR (D), and virus titer determinations (E) were performed to detect IRE1 and PHEV N. (F to K) N2a cells were treated with the indicated concentrations of 4μ8c and kept at the same concentration after PHEV infection. Cells were harvested at 24 h postinfection (hpi). (F) Cytotoxicity assay of 4μ8c in N2a cells. (G) *Xbp1* mRNA splicing assay. (H) Ratios of *Xbp1s*/*Xbp1u* and mRNA expression of the *ERdj4* and *p^58^*^(^*^IPK^*^)^ genes in 4μ8c-treated cells. (I to K) Western blotting (I), qRT-PCR (J), and TCID_50_ assays (K) were performed to detect the PHEV N protein, gene copy numbers, and virus titers in 4μ8c-treated cells. (L to O) Recombinant HA-XBP1s and an empty vector were transiently transfected into N2a cells for 24 h, and the cells were infected with PHEV. At 24 hpi, *Xbp1* mRNA splicing was determined (L), Western blotting was performed to detect the HA and PHEV N proteins (M), the PHEV N gene copy number was calculated by qRT-PCR (N), and the virus titer was determined by a TCID_50_ assay (O). Three independent experiments were performed, and the data are shown as the means ± SD. *, *P* ≤ 0.05; **, *P* ≤ 0.01.

Since all three UPR signaling pathways were activated in response to PHEV infection (Fig. 3 and 4), we next wanted to address which branch of UPR signaling primarily accounted for the inhibition of PHEV replication. In order to elucidate the function of the IRE1-XBP1 axis in PHEV replication, small interfering RNA (siRNA) knockdown and overexpression techniques were employed by transfecting cells with siRNAs specific for IRE1 or recombinant hemagglutinin (HA)-tagged XBP1s eukaryotic expression plasmids, respectively. The IRE1 siRNA knockdown efficiency was measured using qRT-PCR, and siRNA 1 exhibited the best interference efficiency 72 h after transfection (Fig. 6A). IRE1 knockdown inhibited *Xbp1* splicing (Fig. 6B); however, no significant differences in PHEV N protein levels (Fig. 6C), RNA levels (Fig. 6D), and virus titers (Fig. 6E) were observed. To further clarify these findings, we examined the impacts of 4μ8C, a specific inhibitor of IRE1, on viral replication (44). 4μ8C inactivates the active site of IRE1 and blocks *Xbp1* splicing and downstream *ERdj4* and *p^58^*^(^*^IPK^*^)^ induction (Fig. 6F to H). As expected, IRE1 inhibition by 4μ8C did not influence PHEV replication (Fig. 6I to K). Next, HA-XBP1s and the control vector were transiently transfected into N2a cells for 24 h and then infected with PHEV. HA-XBP1s overexpression significantly increased the ratio of *Xbp1s*/*Xbp1u* (Fig. 6L); however, no substantial change in PHEV replication was observed after XBP1s overexpression compared with that of the empty vector (Fig. 6M to O). These findings revealed that the IRE1-XBP1 axis was not involved in PHEV replication.

**FIG 7:**
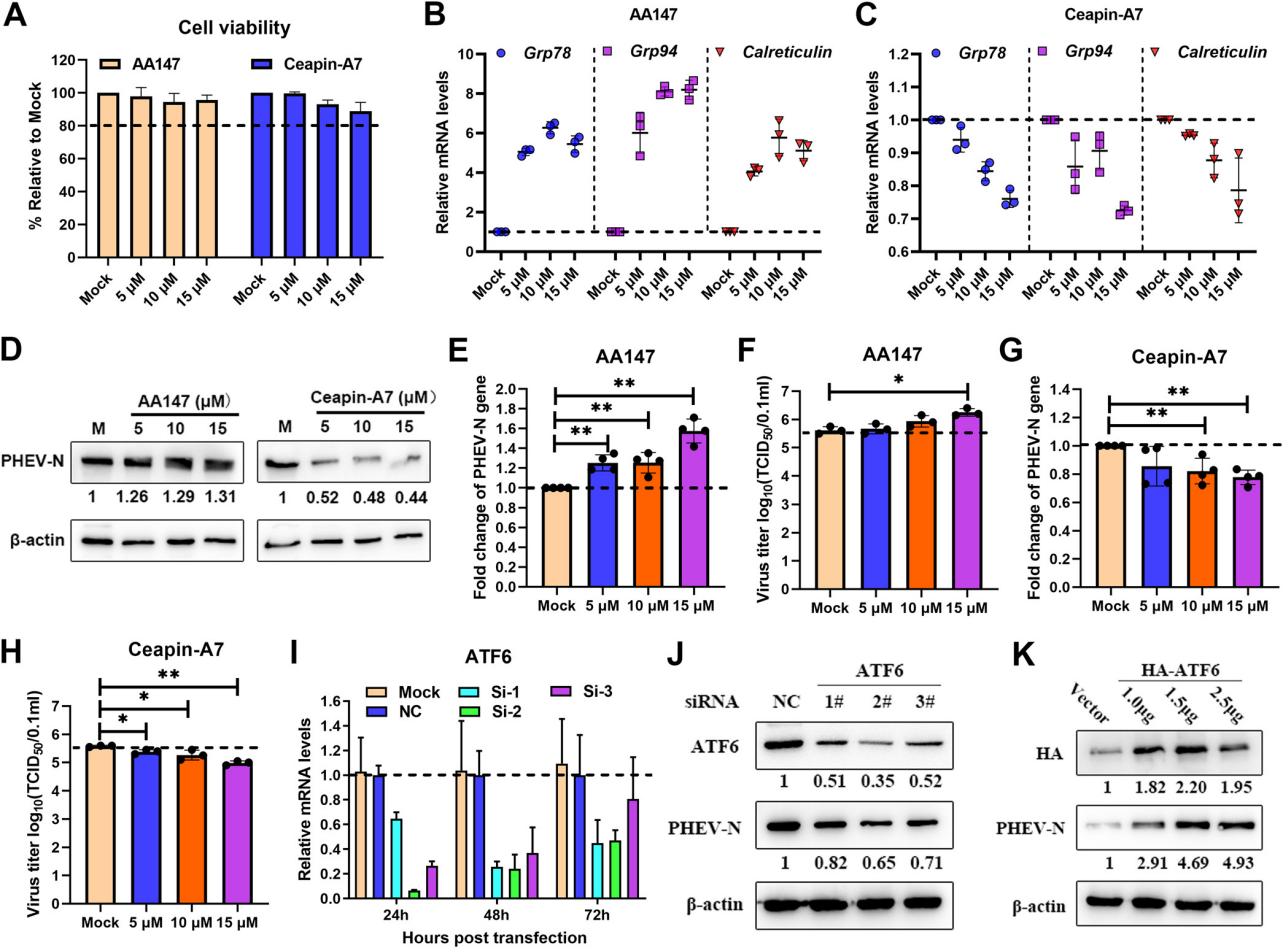
The ATF6 UPR pathway promotes PHEV replication. PHEV-infected N2a cells were treated with the ATF6 activator AA147 (5, 10, and 15 μM) or inhibitor Ceapin-A7 (5, 10, and 15 μM). After virus adsorption, the chemicals were added to the cells immediately and maintained in the medium until cells were harvested at 24 hpi. (A) Cytotoxicities of AA147 and Ceapin-A7 were determined by CCK-8 assays. (B and C) qRT-PCR of *Grp78*, *Grp94*, and *Calreticulin* mRNAs of PHEV-infected N2a cells treated with AA147 (B) and Ceapin-A7 (C). (D) Western blot analysis of PHEV N protein in AA147- and Ceapin-A7-treated and PHEV-infected N2a cells. (E and F) qRT-PCR of the PHEV N gene (E) and virus titers (F) in N2a cells treated with AA147. (G and H) qRT-PCR of the PHEV N gene (G) and virus titers (H) in N2a cells treated with Ceapin-A7. N2a cells were transfected with siRNA specific for ATF6 for 24 h, 48 h, and 72 h. (I) qRT-PCR was used to determine the best interference efficiency. (J and K) N2a cells were transfected with ATF6 siRNA or transiently transfected with the HA-ATF6 overexpression vector for 24 h and then infected with PHEV. At 24 hpi, cells were harvested for Western blotting using antibodies specific for ATF6, HA, and PHEV N protein, and β-actin was used as a sample loading control. The protein band intensities of PHEV N, ATF6, and HA were normalized by β-actin and are given relative to the mock. Three independent experiments were performed, and representative photographs are shown. Data are shown as the means ± SD.

To evaluate the function of the ATF6 branch in PHEV replication, pharmacological manipulation of this pathway was performed using AA147 (selective activator of ATF6) and Ceapin-A7 (a selective ATF6 inhibitor that prevents ATF6 trafficking from the ER to the Golgi apparatus). First, the cell viability of N2a cells after AA147 and Ceapin-A7 treatment was determined using a CCK-8 assay, and the results showed that these two drugs had no obvious cytotoxicity (Fig. 7A). Subsequently, we determined the pharmacological effects of these two drugs on the ATF6 branch in cells infected with PHEV for 24 h at a multiplicity of infection (MOI) of 1 (Fig. 7B and C). AA147 is a small-molecule ER proteostasis regulator that selectively activates the ATF6 arm of the UPR. As expected, AA147 treatment increased the mRNA levels of *Grp78*, *Grp94*, and *Calreticulin* (Fig. 7B), with modest but significant increases of viral N genomic RNA levels, N protein levels, and virus titers (Fig. 7D to F). Ceapin-A7 specifically inhibits ATF6 activation by preventing ATF6 trafficking from the ER to the Golgi apparatus. After Ceapin-A7 treatment, the induction of ATF6 target genes (*Grp78*, *Grp94*, and *Calreticulin*) was reduced (Fig. 7C). Moreover, we found reductions in viral N RNA levels, protein levels, and virus titers in Ceapin-A7-treated and PHEV-infected N2a cells (Fig. 7D, G, and H). These results indicated that the ATF6 branches of the UPR have a proviral role in PHEV infection. In order to further test our hypothesis, a time course knockdown assay was conducted using ATF6-specific siRNA duplexes. The use of qRT-PCR to validate the knockdown performance of ATF6 showed that 24 h after transfection, the optimal knockdown efficiency was obtained (Fig. 7I). Therefore, after 24 h of transfection, N2a cells were infected with PHEV, and Western blotting was performed. ATF6 knockdown decreased PHEV N protein levels (Fig. 7J), indicating that the ATF6 pathway facilitated PHEV replication. To further confirm the function of ATF6 in PHEV replication, HA-ATF6 and the control vector pCMV-HA were transfected into N2a cells, and the cells were then infected with PHEV. The level of PHEV N protein increased after ATF6 was overexpressed (Fig. 7K). These findings showed that the UPR ATF6 pathway promoted rather than inhibited PHEV replication.

### The activated PERK-eIF2α pathway inhibits PHEV replication.

**FIG 8:**
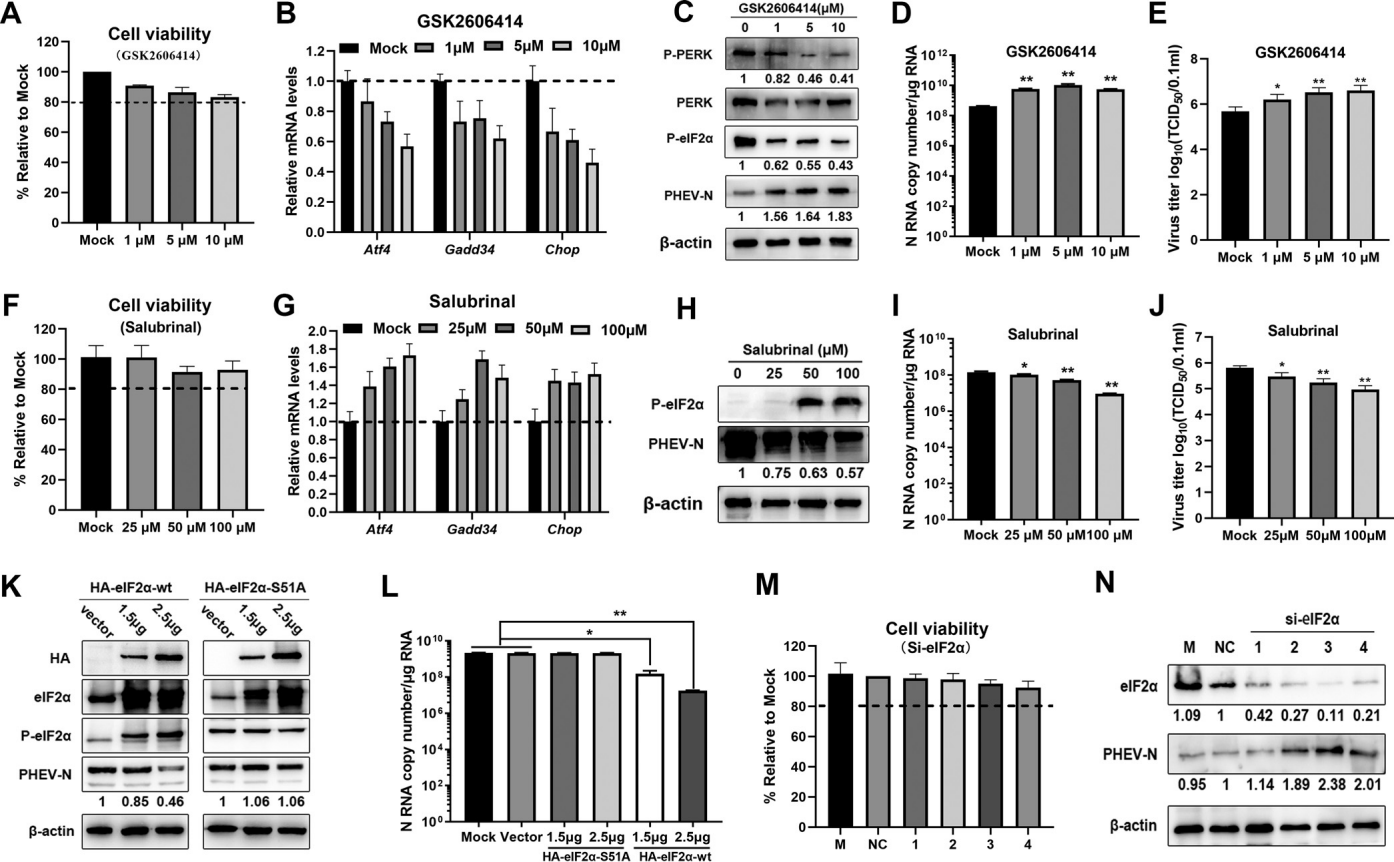
The virus-activated PERK-eIF2α pathway inhibits PHEV replication. (A to E) Disruption of PERK promoted PHEV replication in N2a cells. To disrupt PERK, N2a cells were pretreated with GSK2606414 12 h prior to infection and maintained at that concentration after infection. Cells were collected at 24 hpi. (A and B) Cell viability assay (A) and qRT-PCR analysis of PERK pathway downstream genes (*Atf4*, *Chop*, and *Gadd34*) (B). (C to E) Western blotting (C), qRT-PCR (D), and TCID_50_ assays (E) were conducted to measure PHEV replication. (F to J) Inhibition of eIF2α dephosphorylation by salubrinal suppressed PHEV replication. N2a cells were pretreated with salubrinal at the indicated concentrations 12 h prior to infection and then infected with PHEV in the presence of salubrinal. (F and G) Cell viability assay (F) and qRT-PCR analysis of PERK pathway downstream genes (*Atf4*, *Chop*, and *Gadd34*) (G). (H) At 24 hpi, cell lysates were analyzed by Western blotting using anti-p-eIF2α and anti-PHEV N protein antibodies. (I) PHEV N gene copy numbers were calculated by qRT-PCR. (J) Virus titers were determined by a TCID_50_ assay. (K and L) N2a cells were transfected for 24 h with the eukaryotic expression vectors HA-eIF2α-wt and HA-eIF2α-S51A or the control vector pCMV-HA and then infected with PHEV. At 24 hpi, Western blotting was conducted using antibodies against HA, eIF2α, p-eIF2α, PHEV N, or β-actin (K), and N gene copy numbers were calculated by qRT-PCR (L). (M and N) N2a cells were transfected with eIF2α or NC siRNA for 24 h and subsequently infected with PHEV. (M) Cell viability was determined by a CCK-8 assay. (N) At 24 hpi, eIF2α and PHEV N were analyzed by Western blotting. Three independent experiments were performed, and data are shown as the means ± SD. *, *P* ≤ 0.05; **, *P* ≤ 0.01.

In order to clarify the effects of the PERK-eIF2α axis of the UPR on PHEV replication, GSK2606414, a specific PERK inhibitor, was initially used to disrupt PERK activity. First, cell viability and the inhibitory effect of GSK2606414 were determined, and the results showed that the concentration of GSK2606414 had obvious inhibitory efficiency against PERK from the decrease in *Atf4*, *Chop*, and *Gadd34* transcription with no cytotoxicity (Fig. 8A and B). Subsequently, Western blotting with antibodies specific for p-PERK and p-eIF2α was performed to further confirm the PERK inhibition efficiency (Fig. 8C). As expected, we observed that GSK2606414 treatment reduced the phosphorylation of PERK and eIF2α with increased PHEV N protein in N2a cells (Fig. 8C), and the increase of the N protein content correlated with N RNA and virus production (Fig. 8D and E), indicating the antiviral effect of PERK-eIF2α on PHEV replication.

Next, N2a cells were pretreated for 6 h with various concentrations of salubrinal, a specific eIF2α dephosphorylation inhibitor, and then infected with PHEV at an MOI of 1, with salubrinal being maintained in the medium until cells were harvested at 24 hpi. Cell viability assays showed that the concentration of salubrinal had no cytotoxicity (Fig. 8F). The inhibition of eIF2α dephosphorylation was confirmed by measuring the induction of eIF2α target genes (*Atf4*, *Chop*, and *Gadd34*) (Fig. 8G). Salubrinal treatment resulted in declines in PHEV N protein and RNA, and dose-dependent reductions in PHEV N protein and RNA were seen at different salubrinal concentrations (Fig. 8H and I). This was confirmed by a 50% tissue culture infectious dose (TCID_50_) assay, which demonstrated that the contents of PHEV N protein and RNA strictly correlated with virus titers (Fig. 8J). These findings showed that the PERK-eIF2α axis of the UPR negatively regulated PHEV replication.

To further investigate the function of eIF2α in the regulation of PHEV replication, we examined PHEV N protein and mRNA levels in N2a cells after transfection with wild-type (wt) eIF2α (HA-eIF2α-wt) and an unphosphorylatable eIF2α mutant variant with Ala taking the place of Ser at residue 51 (HA-eIF2α-S51A). While PHEV replication was inhibited in eIF2α-wt-transfected cells, no reduction in PHEV replication was observed in eIF2α-S51A-transfected cells (Fig. 8K and L). In addition, eIF2α was knocked down by specific siRNA, and cell viability was determined given the essential function of eIF2α in protein translation. The results showed that partial interference with eIF2α function did not significantly affect cell viability (Fig. 8M). As expected, a significant increase in PHEV replication was observed in eIF2α knockdown cells (Fig. 8N). Overall, these results show that the UPR sensor PERK suppresses PHEV replication by phosphorylating eIF2α and that Ser at residue 51 is a key phosphorylation site.

### PKR is activated by dsRNA during PHEV infection and inhibits PHEV replication by phosphorylating eIF2α.

**FIG 9:**
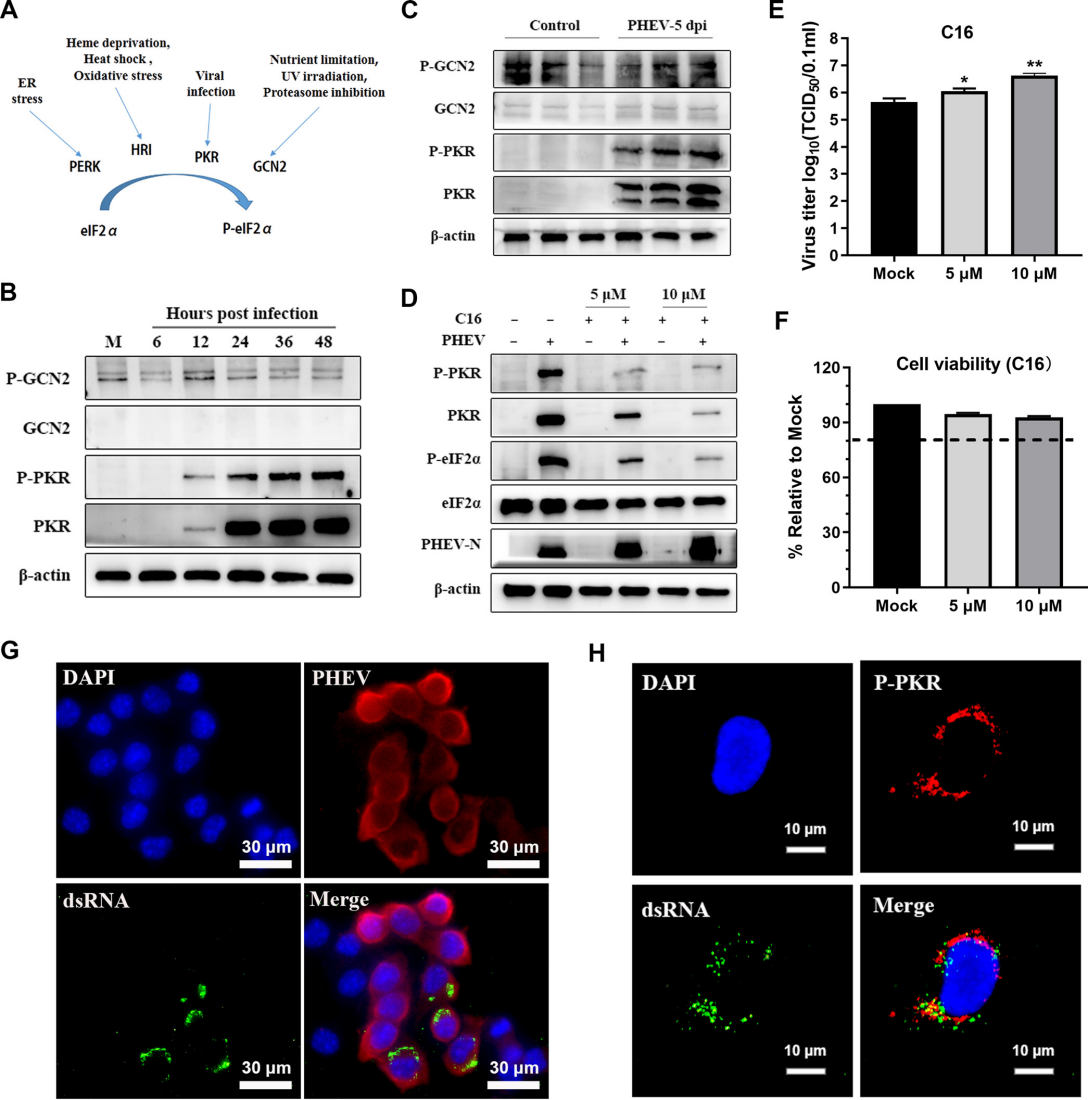
Induction of other eIF2α kinases during PHEV infection. (A) Diagram of four known eIF2α kinases that regulate eIF2α activities via eIF2α phosphorylation under different stress conditions. (B and C) PKR but not GCN2 was activated during PHEV infection. Mock-infected or PHEV-infected N2a cells (B) or mouse brains (C) were harvested for Western blot analysis with antibodies against GCN2, p-GCN2, PKR, and p-PKR. (D) Inhibition of PKR by C16 promoted PHEV replication. N2a cells were pretreated with the indicated concentrations of C16 for 2 h and then infected with PHEV for 24 h in the presence of C16. Western blotting was performed with anti-PKR, -p-PKR, -eIF2α, -p-eIF2α, -PHEV N, and -β-actin antibodies. (E) Virus titers were measured in N2a cells infected with PHEV (MOI of 1) for 24 h in the presence or absence of C16. (F) N2a cells were treated with the indicated concentrations of C16, and cell viability was measured using a CCK-8 assay. (G) dsRNA was produced in PHEV-infected N2a cells. N2a cells were infected with PHEV for 24 h, and immunofluorescence staining was performed using anti-dsRNA and anti-PHEV N antibodies. (H) dsRNA was colocalized with p-PKR. N2a cells were infected with PHEV for 24 h, and immunofluorescence staining was performed using anti-dsRNA and anti-p-PKR antibodies. Three independent experiments were performed, and data in panels E and F are shown as the means ± SD. *, *P* ≤ 0.05; **, *P* ≤ 0.01.

In addition to PERK, the other three eIF2α phosphorylation kinases are PKR, heme-regulated inhibitor (HRI), and general control nonderepressible 2 (GCN2), and the downstream reactions induced by these four kinases are collectively termed the integrated stress response (ISR) (Fig. 9A) (45). Considering that the eIF2α phosphorylation kinases associated with virus infection include PKR and GCN2 (42, 46), we examined the status of PKR and GCN2 after PHEV infection both *in vitro* and *in vivo*. In PHEV-infected N2a cells, we observed that PKR and p-PKR, but not GCN2 and p-GCN2, were progressively increased and peaked at 48 hpi (Fig. 9B). Consistent with the results *in vitro*, compared with control mice, PHEV infection activated PKR but not GCN2 *in vivo* (Fig. 9C). To verify the role of PKR in eIF2α phosphorylation and PHEV replication, C16, a specific inhibitor of PKR, was utilized to disrupt the PKR signaling pathway (43). PKR inhibition was confirmed by Western blotting for p-PKR and p-eIF2α (Fig. 9D). C16 treatment substantially increased PHEV replication in a dose-dependent manner, as shown by PHEV N protein levels and viral titers, and was associated with the inhibition efficacy of p-PKR and p-eIF2α (Fig. 9D and E). The PHEV replication enhancement was not due to an increase in cell proliferation because C16 treatment did not alter cell viability (Fig. 9F). Immunofluorescence results showed that double-stranded RNA (dsRNA) was generated in PHEV-infected N2a cells and colocalized with p-PKR (Fig. 9G and H), suggesting that dsRNA was generated as a by-product during PHEV genome replication and interacted with PKR (47). These results reveal that PKR also accounted for eIF2α phosphorylation and the negative regulation of PHEV replication, at least in part.

### The PERK/PKR-eIF2α pathway inhibits PHEV replication by attenuating global protein translation.

**FIG 10:**
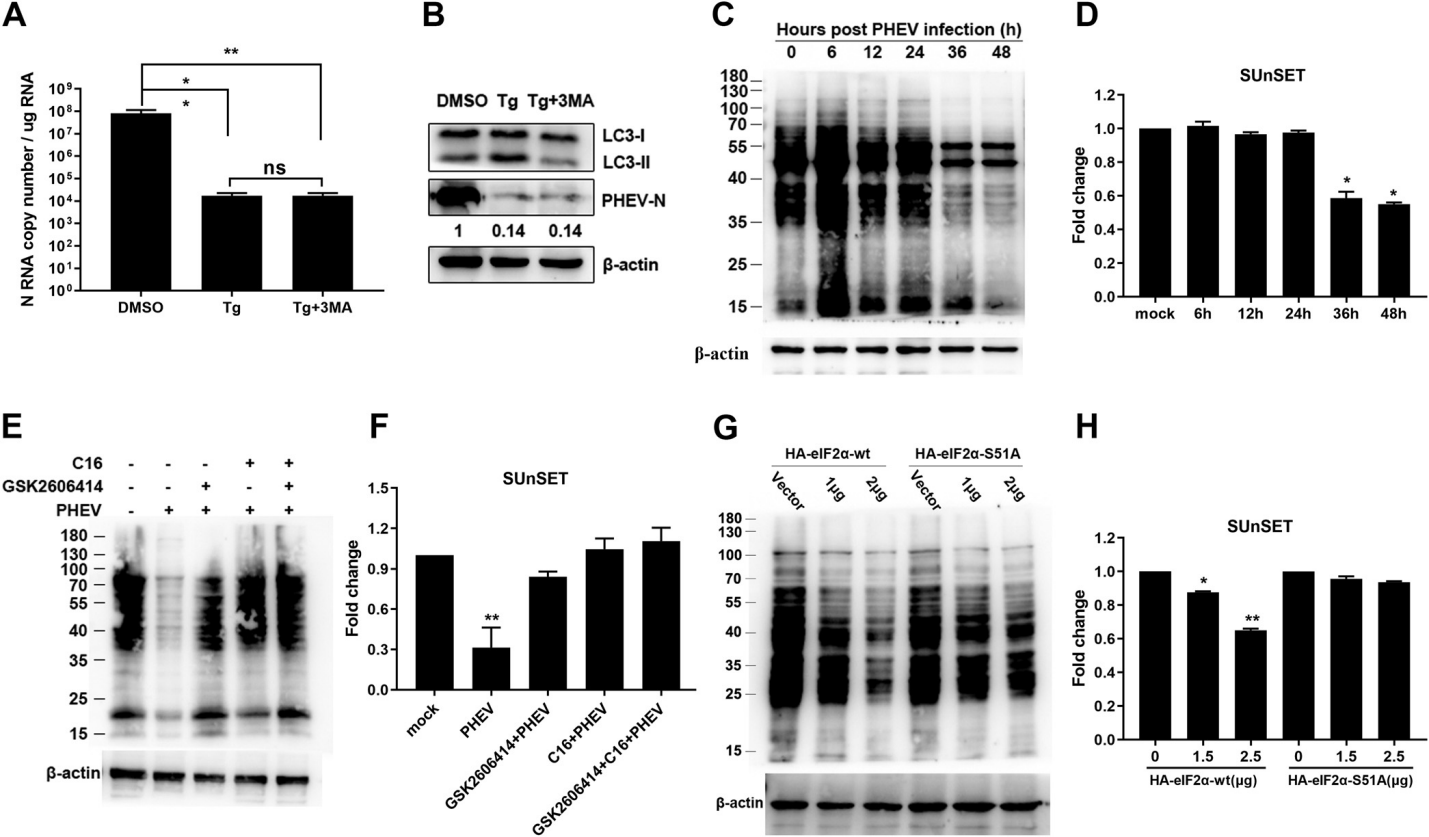
Analysis of global protein translation by the SUnSET assay. (A and B) Autophagy is irrelevant to PERK/PKR-eIF2α-mediated PHEV replication inhibition. N2a cells were pretreated 2 h before infection with Tg alone or as a mixture with 3-MA and retained at the same concentration after infection. N RNA copy numbers were calculated by qRT-PCR (A), and LC3 and PHEV N expression was determined by Western blotting (B). (C and D) PHEV infection attenuated global protein translation. N2a cells were mock infected or infected with PHEV for 6, 12, 24, 36, or 48 h before SUnSET analysis. (C) Immunoblotting was performed with an antipuromycin antibody, and β-actin served as a sample loading control. (D) Fold changes in the band intensity and statistical analyses. The value for mock-infected cells was set to 1.0. (E and F) Inhibition of PERK and PKR by GSK2606414 and C16 alone or in combination ameliorated PHEV-induced translation attenuation. N2a cells were pretreated with GSK2606414 and C16 separately or in combination 2 h before infection and maintained the same concentration after infection. Immunoblotting (E) and fold changes (F) are shown. (G and H) N2a cells were analyzed for ongoing translation after transfection with HA-eIF2α-wt or HA-eIF2α-S51A overexpression vectors. For immunoblot analysis, three independent experiments were performed, and representative images are shown. Data in panels A, D, F, and H are shown as the means ± SD. *, *P* ≤ 0.05; **, *P* ≤ 0.01; ns, not significant.

Next, we wanted to investigate how the PERK/PKR-eIF2α pathways inhibited PHEV replication. Several cellular modifications, including apoptosis, autophagy, and reversible attenuation of global protein translation, are theoretically triggered by the phosphorylation of eIF2α. A previous study demonstrated that PHEV infection induces atypical autophagy and that autophagy negatively regulates PHEV replication (48). However, pretreatment of N2a cells with 3-methyladenine (3-MA), a commonly utilized autophagic inhibitor, failed to rescue the Tg-mediated PHEV replication reduction (Fig. 10A and B), suggesting that autophagy was not related to p-eIF2α-mediated inhibition of PHEV replication. Phosphorylated eIF2α halts translation initiation and reduces global protein translation (49). To investigate whether p-eIF2α-mediated translation attenuation was responsible for PHEV inhibition by the PERK/PKR-eIF2α pathway, we used surface sensing of translation (SUnSET), a nonradioactive approach, to monitor nascent protein synthesis in PHEV-infected N2a cells, as mentioned previously (50). From 36 to 48 h after infection, puromycin-labeled proteins were substantially decreased by 40% to 50% (Fig. 10C and D), indicating that PHEV significantly inhibited the total protein translation levels in the late stage of infection.

To further validate that the reduced nascent protein synthesis following PHEV infection was involved in PERK/PKR-triggered eIF2α phosphorylation, GSK2606414 (a PERK-specific inhibitor) and C16 (a PKR-specific inhibitor) were used to investigate protein translation (43). The addition of GSK2606414 and C16 alone or in combination rescued the global protein translation attenuation of PHEV infection (Fig. 10E and F). These results were further verified by the overexpression of eIF2α in N2a cells. By using the SUnSET method, a reduction in nascent protein translation was observed in wild-type eIF2α (HA-eIF2α-wt)-transfected cells but not in cells transfected with the unphosphorylated form of eIF2α (HA-eIF2α-S51A) (Fig. 10G and H). These findings indicated that the inhibition of PHEV replication by the PERK/PKR-eIF2α pathway occurred at least in part by the attenuation of global protein translation induced by eIF2α phosphorylation.

### Phosphorylated eIF2α promotes stress granule formation to suppress PHEV replication.

**FIG 11:**
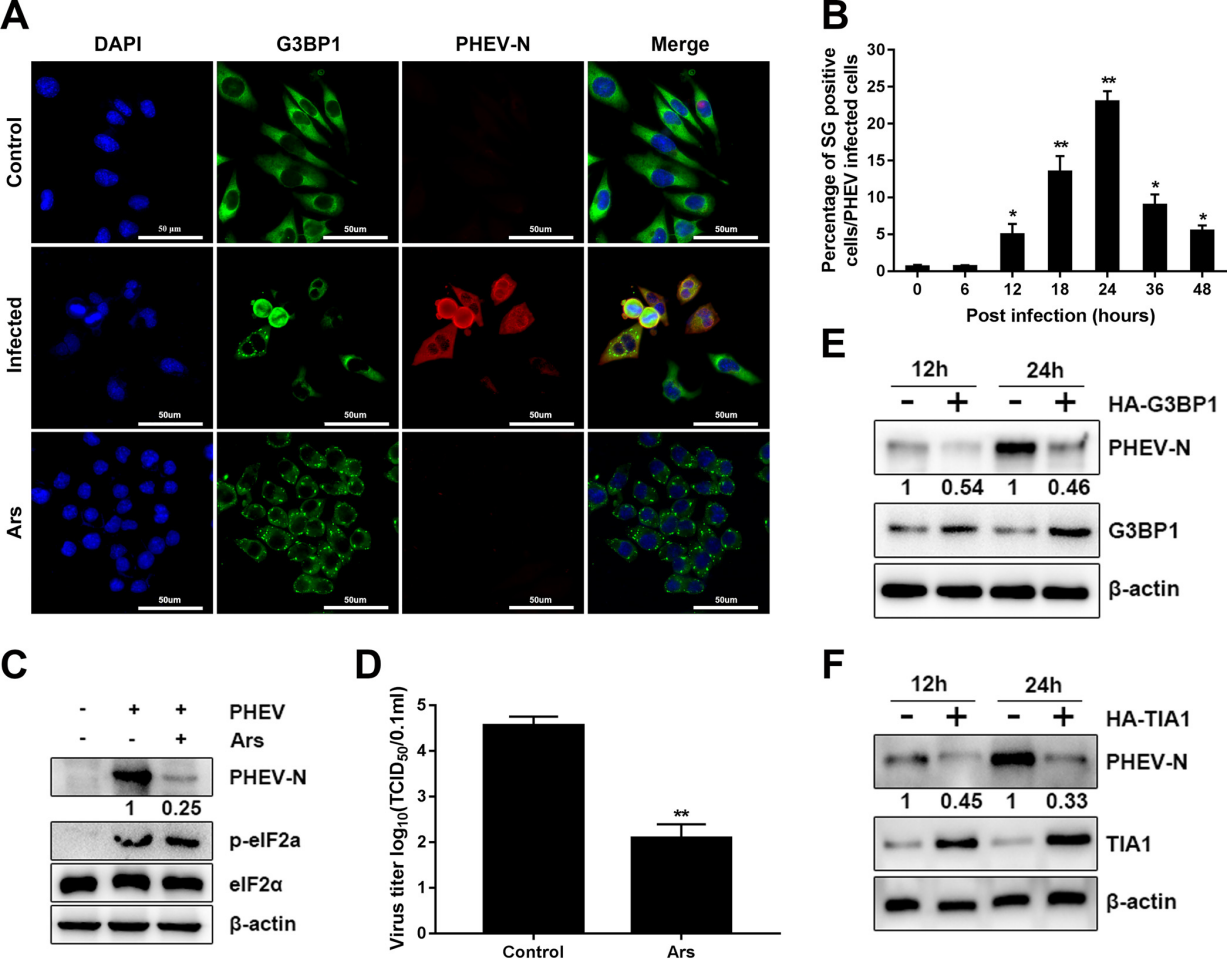
SG formation induced by PHEV infection inhibits virus replication in N2a cells. (A and B) PHEV induces transient SG formation in N2a cells during the late stage of infection. N2a cells were mock infected or infected with PHEV (MOI of 1) for 6, 12, 18, 24, 36, and 48 h; fixed; and then stained with anti-G3BP1 and -PHEV N antibodies for immunofluorescence analysis. Mock-infected cells were treated with DMEM, and cells were treated with 0.5 mM Ars for 1 h as a positive control. (A) Representative images of SGs. (B) Percentage of SG-positive cells in PHEV-infected cells (approximately 200 cells). (C and D) SGs induced by Ars inhibit PHEV biosynthesis. N2a cells were treated with 0.5 mM Ars for 1 h and then infected with PHEV (MOI of 1) for 12 h. Cells were harvested for Western blotting (C) and virus titer determinations (D). (E and F) Overexpression of SG proteins inhibits PHEV replication. N2a cells were overexpressed with G3BP1, TIA1, and an empty vector control. After 24 h of transfection, cells were infected with PHEV (MOI of 1) and collected at 12 and 24 hpi for Western blotting. Three independent experiments were performed. Data in panels B and D are shown as the means ± SD. *, *P* ≤ 0.05; **, *P* ≤ 0.01.

Given that the p-eIF2α can induce SG formation and the significant functions of SG in virus-induced stress responses and antiviral innate immunity (29), we examined whether PHEV infection induced SG formation in infected cells. N2a cells were infected with PHEV at an MOI of 1 for the indicated times, and an indirect immunofluorescence assay was performed using antibodies against G3BP1 and PHEV N, with arsenite (Ars)-treated cells serving as a positive control. Immunofluorescence microscopy revealed that the cytoplasm of PHEV-infected and Ars-treated cells but not uninfected cells contained G3BP1-positive particles (Fig. 11A), indicating that SGs were formed in PHEV-infected cells. The kinetics of SG formation in PHEV-infected cells, however, were delayed until 12 h postinfection, peaked at 24 h postinfection, and sharply diminished thereafter (Fig. 11B), implying that PHEV induced transient SG formation during the later stage of infection.

Phosphorylation of eIF2α is the key pathway of typical SG formation induced by Ars. To investigate the function of virus-induced SGs in PHEV replication, N2a cells were pretreated for 1 h with Ars and then infected with PHEV, and cells were harvested at 12 hpi for Western blotting, qRT-PCR, and virus titer determination. In comparison to the controls, neither PHEV infection nor Ars treatment had any effect on eIF2α levels (Fig. 11B). However, the levels of phosphorylated eIF2α were dramatically elevated in both virus-infected cells and Ars-treated PHEV-infected cells. Furthermore, PHEV N protein levels were considerably lower in Ars-treated cells than in cells not treated with Ars (Fig. 11C). PHEV suppression by Ars was further confirmed by virus titer determination (Fig. 11D). These findings suggested that PHEV replication was inhibited by preformed SGs (induced by Ars) in host cells.

To further clarify the functions of SGs in PHEV replication, N2a cells were infected with PHEV at an MOI of 1 after overexpressing G3BP1 or TIA1, and Western blotting was performed at 12 and 24 h. G3BP1 and TIA1 are the key elements of SGs, and the overexpression of the two proteins induces the assembly of cytosolic SGs. Our results revealed that the expression levels of viral protein were considerably suppressed following G3BP1 and TIA1 overexpression (Fig. 11E and F). These findings showed that virus-induced SGs adversely regulated PHEV replication and possessed potential antiviral capabilities.

## DISCUSSION

ERS and the downstream UPR are generally engaged in coronavirus replication and modulate the innate immune response of the host (24–26, 37, 40, 51–54). Nearly all coronaviruses have the potential to cause ERS in host cells, which is mainly related to their viral characteristics. First, the processing, folding, and glycosylation modification of newly synthesized viral proteins pose a heavy burden to the ER. Second, the progeny virus “buds” in the ER-Golgi intermediate compartment to release progeny viral particles, resulting in the extensive depletion of ER lipid membrane components and morphological rearrangement (51). A large amount of evidence indicates that coronavirus infection causes ERS and triggers the UPR, thus regulating virus replication and proliferation (27, 55).

In this study, all three UPR branches and the PKR-eIF2α pathway were activated both *in vitro* and *in vivo* following PHEV infection. However, each pathway of the three UPR branches has a different role in viral replication. The IRE1α pathway was found to be irrelevant to PHEV replication, according to the results of siRNA interference, pharmacological treatment, and overexpression assays (Fig. 6). Interestingly, the ATF6 pathway was favorable for viral replication by using the ATF6 activator AA147 and inhibitor Ceapin-A7 (Fig. 7A to F). Moreover, specific siRNA knockdown of the ATF6 pathway decreased PHEV replication, while the overexpression of ATF6 increased PHEV replication (Fig. 7G to I), indicating that the ATF6 branch of the UPR facilitated viral replication. A previous study demonstrated that African swine fever virus (ASFV) infection triggers the ATF6 signaling pathway to facilitate viral replication by preventing early apoptosis (56). ATF6 is also necessary for effective West Nile virus (WNV) replication by promoting cell survival and inhibiting host innate immune responses (57). However, we were unable to detect an obvious trimmed version of ATF6 or clear nuclear translocation in both PHEV-infected and positive-control (Tu- and Tg-treated) N2a cells; thus, a further-detailed mechanism of ATF6 in regulating PHEV replication is worthwhile and necessary.

The PERK-eIF2α pathway is strongly linked to viral replication and pathogenesis (58–60). Our results here show that the PERK-eIF2α axis was activated following PHEV infection and negatively regulated PHEV replication through global protein translation attenuation. Similar to this finding, the replication of PEDV and TGEV is also suppressed by PERK signaling (27, 41). In contrast, for infectious bronchitis virus (IBV) and mouse hepatitis virus (MHV), replication was not markedly affected by activated PERK-eIF2α (26, 39, 59). However, in a recent article, the authors claimed that inhibition of the PERK-eIF2α-ATF4 pathway by GSK2606414 and integrated stress response inhibitor (ISRIB) was able to reduce MHV replication, indicating that the activation of this pathway had a proviral role (55). Thus, PERK-eIF2α signaling has diverse roles in different coronavirus infections, and the detailed mechanism needs to be further clarified. The three UPR signaling pathways are integrated systems and engage in complex cross talk, and the inhibitory effect of ERS on PHEV is the result of the synergistic effects of these pathways. Aside from PERK, there are three other eIF2α kinases that have been identified currently, PKR, GCN2, and HRI (45). In this study, we found that PKR, but not GCN2, was activated by dsRNA produced by PHEV infection and partly accounted for the inhibition of PHEV replication through the phosphorylation of eIF2α. These results correspond to the results of a previous report that showed that TGEV infection activates PKR, leading to an increase in eIF2α phosphorylation and a shutdown of host translation (61).

To reestablish ER homeostasis in the face of ERS, phosphorylated eIF2α reduces global protein translation by impeding translation initiation, together with the translation of mRNAs encoding bZIPs such as *Atf4*, reducing the protein loads in the ER lumen and increasing the capacity of the ER to handle unfolded proteins to effectively manage stress conditions (20, 49). Here, our results show that PHEV infection caused considerable phosphorylation of eIF2α, which in turn resulted in an obvious reduction in global protein translation, including viral protein translation (Fig. 10C to H). Generally, p-eIF2α-mediated global protein translation attenuation represents a host defense response to viral infection and does not discriminate between viral and host mRNAs, especially viruses that use the same translation strategies as eukaryotic cells. As a result, many viruses have evolved countermeasures. For example, TGEV protein 7 accelerates eIF2α dephosphorylation by interacting with the catalytic subunit of protein phosphatase 1 (PP1c) to counteract host defense (61). In this study, phosphorylated eIF2α accumulated 12 h after PHEV infection, but significant inhibition of global protein translation was observed after 24 h of PHEV infection (Fig. 3C and Fig. 10C and D). Thus, further analyses should be performed to clarify whether any PHEV products act to counteract p-eIF2α-mediated translation inhibition.

Phosphorylated eIF2α is closely linked to SG formation (62). According to a previous study, SGs have important functions in the host antiviral response to various viral infections (30, 31, 63, 64). Moreover, several viruses have evolved strategies to counteract the antiviral impact of SGs, such as cleavage of G3BP1 by viral protease or sequestration of TIA1 within viral inclusions to prevent SG formation (65–67), suggesting that viruses may modulate SGs to regulate the host antiviral response for their own benefit. Here, we found that PHEV induced transient SG formation in the later stage of infection, with approximately 25% of PHEV-infected N2a cells displaying two or more SGs at 24 hpi, followed by a rapid decrease in SG formation. No cleaved G3BP1 products were identified during PHEV infection (data not shown). Moreover, the replication of PHEV was hindered by Ars-induced SGs and G3BP1 and TIA1 overexpression in N2a cells. Similar observations were also reported for the alphacoronavirus PEDV (32, 65). Further investigations are needed to further identify the detailed interaction networks in SG formation and PHEV replication.

**FIG 12:**
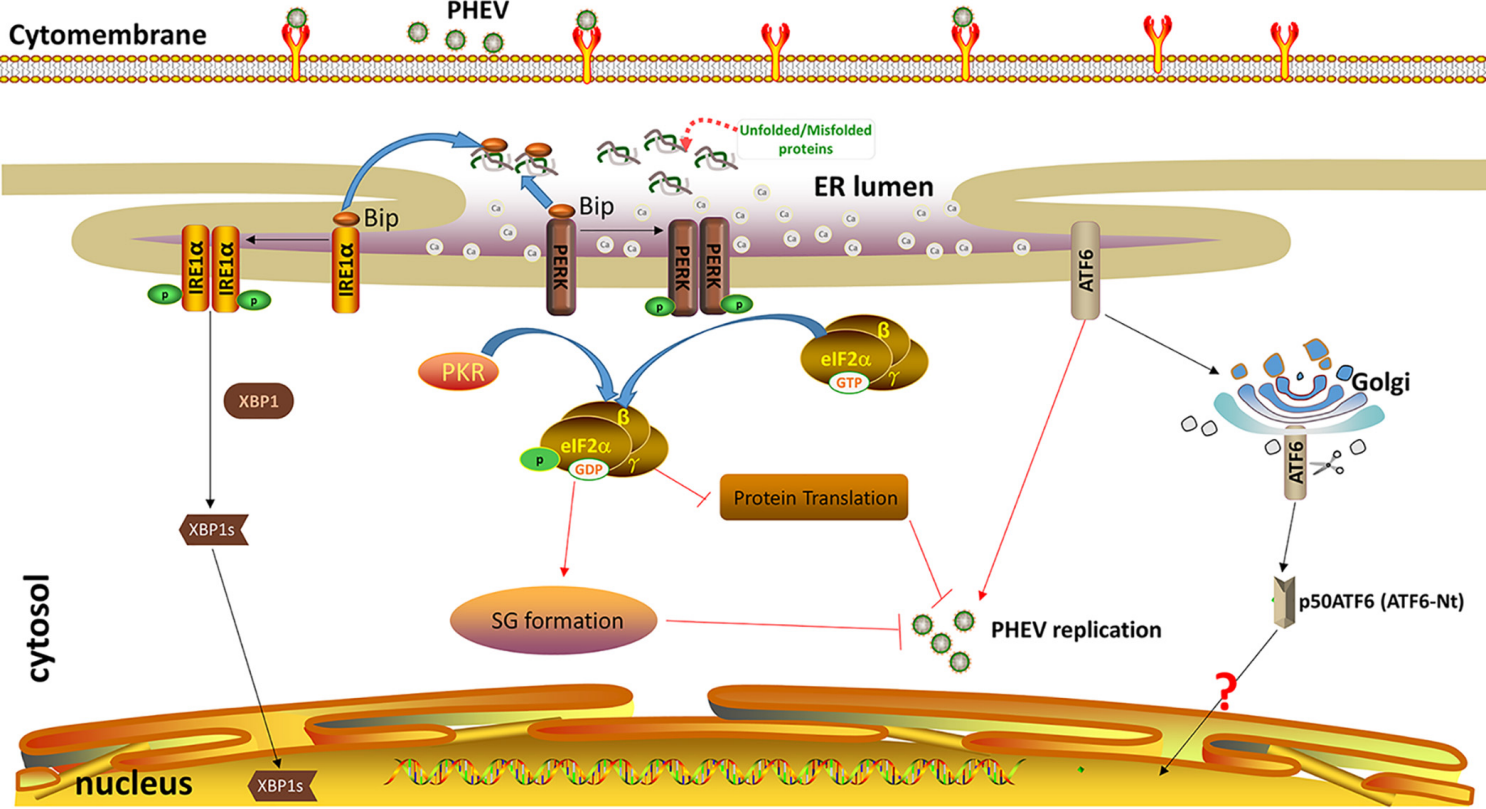
Model of PERK/PKR-eIF2α negative regulation of PHEV replication. PHEV infection induces ER stress both *in vitro* and *in vivo*, and host cells sense PHEV infection and induce the activation of ATF6, IRE1, and PERK of the UPR and PKR of the ISR. Activated PERK and PKR activate eIF2α by phosphorylating serine at residue 51, and p-eIF2α negatively regulates PHEV replication by attenuating global protein translation and SG formation. The red arrows and the blunt-ended lines indicate activation and inhibition, respectively.

Collectively, these findings show that PHEV infection changed the ER morphology, triggered ERS, and activated all three UPR branches, with the activated PERK pathway predominantly accounting for PHEV replication inhibition. Moreover, the PKR-eIF2α pathway was also activated by dsRNA following PHEV infection. PERK and PKR acted collaboratively to suppress PHEV replication by inhibiting protein translation and facilitating SG formation (Fig. 12). Further research is needed to clarify the detailed mechanism by which SGs suppress viral replication. These findings enrich our knowledge of the virus-cell interaction and may provide new antiviral targets for the treatment of PHEV and other coronavirus infections.

## MATERIALS AND METHODS

### Ethics statement.

All mouse experiments were approved by the Institutional Animal Care and Use Committee (IACUC) of Jilin University. In animal experiments, mice were anesthetized under 3.5% chloral hydrate (1.0 mL/100 g; Sigma, USA), and all efforts were made to reduce suffering.

### Animal experiments.

Twenty 3-week-old BALB/c mice were allocated into two groups randomly. Mice in group 1 were intranasally inoculated with 20 μL of 10^4.6^ 50% tissue culture infectious doses (TCID_50_) of PHEV strain CC14 (PHEV-CC14) (GenBank accession number MF083115). Mice in group 2 were intranasally inoculated with 20 μL of Dulbecco’s modified Eagle’s medium (DMEM) as a control. Clinical signs were examined every day, and brains were harvested daily for 5 days for qRT-PCR, Western blotting, and immunofluorescence analysis.

### Cells, viruses, and antibodies.

Mouse neuro-2a (N2a) cells (ATCC CCL-131) were maintained in DMEM (Gibco, USA) supplemented with 10% (vol/vol) fetal bovine serum (FBS) (HyClone) and 1% penicillin-streptomycin in a cell incubator at 37°C with 5% CO_2_. PHEV-CC14 (GenBank accession number MF083115) is a wild-type (wt) virus that was isolated from a naturally infected piglet in Changchun, China, and propagated in N2a cells (8, 68). PHEV was inactivated by exposing the above-mentioned virus stock to a water bath at 56°C for 30 min, and the inactivated viruses were stored at −80°C until use. Antibodies against β-actin (catalog number 66009-1-Ig), GRP78/Bip (catalog number 11587-1-AP), calnexin (catalog number 10427-2-AP), PDI (catalog number 11245-1-AP), IRE1 (catalog number 27528-1-AP), and PKR (catalog number 18244-1-AP) were purchased from Proteintech (Wuhan, China). Purified anti-ATF6 antibody (catalog number 853101) was obtained from BioLegend (San Diego, CA). Antibodies against GRP94 (catalog number 2104), eIF2α (catalog number 5324), and p-eIF2α (Ser51) (catalog number 3398) were purchased from Cell Signaling Technology (CST) (Beverly, MA). Antibodies against p-GCN2 (T899) (catalog number ab75836), p-PERK (catalog number ab192591), and PERK (catalog number ab229912) were purchased from Abcam (Cambridge, MA). A rabbit polyclonal antibody recognizing PKR phosphor (Thr446) (catalog number ARG51761) was purchased from Arigobio (Hamburg, Germany). GCN2 polyclonal antibody (catalog number A12618) was purchased from Abclonal (Wuhan, China). Mouse anti-double-stranded RNA (J2) (catalog number 10010500) was purchased from Nordic-MUbio (Susteren, Netherlands). The antipuromycin monoclonal antibody clone 12D10 (catalog number MABE343) was obtained from Sigma (Millipore, Billerica, MA, USA). PHEV N rabbit polyclonal antibody was prepared and stocked in our laboratory.

### Virus inoculation assay and chemical treatment.

N2a cells were infected with PHEV-CC14 at an MOI of 1, treated with inactivated virus, or mock infected with DMEM; adsorbed at 37°C for 90 min; and washed three times with phosphate-buffered saline (PBS), and the cells were then cultured in DMEM containing 2% FBS at 37°C with 5% CO_2_ for the indicated times. In cell experiments, all the infections were carried out at an MOI of 1. Tunicamycin (Tu) (catalog number MB5419), thapsigargin (Tg) (catalog number MB13319), 4μ8C (catalog number MB3468), and salubrinal (catalog number MB2345) were purchased from Dalian Meilunbio Biotechnology Co., Ltd. (Dalian, China). GSK2606414 (catalog number HY-124293) was obtained from Selleck (Shanghai, China). The imidazole-oxindole PKR inhibitor C16 (catalog number I9785), 4-phenylbutyric acid (4-PBA) (catalog number P21005), puromycin (catalog number P8833), Ceapin-A7 (catalog number SML2330), and sodium arsenite (Ars) (catalog number S7400) were acquired from Sigma-Aldrich (Darmstadt, Germany). AA147 (catalog number HY-124293) was obtained from MedChemExpress (MCE) (Shanghai, China). Detailed information on the drugs employed in this research is presented in Table 1. N2a cells were pretreated with the indicated concentrations of drugs or the same volume of dimethyl sulfoxide (DMSO) for different times, followed by infection with PHEV-CC14. After adsorption for 90 min, the supernatants were replaced with normal cell culture medium containing the chemicals at the indicated concentrations.

**TABLE 1:** Details of all drugs employed in this study^*a*^

Drug	Solvent	Storage temp (°C)	Storage concn	Working concn
Tu	DMSO	−20	1,000 μg/mL	2 μg/mL
Tg	DMSO	−20	2 mM	1 μM
4-PBA	DMSO	−20	1 M	0.5/1/2/4 mM
4μ8C	DMSO	−20	50 mM	50/100 μM
Ceapin-A7	DMSO	−20	10 mM	5/10/15 μM
AA147	DMSO	−20	20 mM	5/10/15 μM
GSK2606414	DMSO	−20	50 mM	1/5/10 μM
Salubrinal	DMSO	−20	50 mM	25/50/100 μM
C16	DMSO	−20	20 mM	1/5/10 μM
3-MA	ddH_2_O	−20	50 mM	10 mM
NaAs (Ars)	ddH_2_O	−20	1 M	0.5 mM

a Tu, tunicamycin; Tg, thapsigargin; 4-PBA, 4-phenylbutyric acid; DMSO, dimethyl sulfoxide; ddH_2_O, double-distilled water.

### Viral titer determination.

Infected cell cultures were collected, freeze-thawed three times, and serially diluted 10-fold from 10^−1^ to 10^−10^ in DMEM. Dilutions were used to inoculate confluent N2a cells in 96-well formats for up to 72 h. Next, the supernatant was discarded, and 100 μL of 4% polyformaldehyde was added to each well to fix the cells. Immunofluorescence analysis was performed using anti-PHEV N antibody to detect viral antigen. The Reed-Muench method was used to calculate the TCID_50_/0.1 mL.

### Transmission electron microscopy analysis.

For transmission electron microscopy (TEM) analysis, mouse brains and N2a cells were either mock infected or infected with PHEV for the indicated times. The samples were collected for thin sectioning and observed using TEM as previously described (69).

### Plasmid construction and transient transfection.

Plasmids pHA-XBP1s, pHA-ATF6, pHA-eIF2α, pHA-G3BP1, and pHA-TIA1 were constructed using conventional cloning techniques. The RT-PCR primers and the corresponding restriction sites (underlined) are as follows: XBP1s-F (5′-GGAAGATCTATGGTGGTGGTGGCAGCG-3′ [BglII]), XBP1s-R (5′-AAAAGCGGCCGCTTAGACACTAATCAGCTGGGGG-3′ [NotI]), eIF2α-wt-F (5′-CCGGAATTCATGCCGGGGCTAAGTTGTAGA-3′ [EcoRI]), eIF2α-wt-R (5′-AAAAGCGGCCGCTTAATCTTCAGCTTTGGCTTCC-3′ [NotI]), ATF6-F (5′-CCGCTCGAGATGGAGTCGCCTTTTAGTCC-3′ [XhoI]), ATF6-R (5′-AAAAGCGGCCGCCTACTGCAACGACTCAGGGAT-3′ [NotI]), G3BP1-F (5′-CGCGGATCCATGGTTATGGAGAAGCCT-3′ [BamHI]), G3BP1-R (5′-ACGCGTCGACTCACTGCCTTGGAGTTGT-3′ [SalI]), TIA1-F (5′-CGCGGATCCATGGAGGACGAGATGCCCAA-3′ [BamHI]), and TIA1-R (5′-ACGCGTCGACTCACTGGGTTTCATACCCGG-3′ [SalI]). The target fragments were inserted into a pCMV-HA vector (Clontech) using the corresponding restriction enzyme cutting sites mentioned above. pHA-eIF2α-S51A that introduces the Ser51Ala substitution for eIF2α was generated using the Fast mutagenesis system (catalog number FM111-01; Transgen, Beijing, China) (P1, 5′-TCTTCTTAGTGAATTAGCCAGACGACGTAT-3′; P2, 5′-CTAATTCACTAAGAAGAATCATGCCTTCAA-3′). The recombinant plasmids were transfected into N2a cells using Lipofectamine 3000 reagent (Invitrogen, Carlsbad, CA).

### RNA interference.

siRNA targeting ATF6 (siATF6), siIRE1, sieIF2α, and the nontargeting control (NC) were obtained from GenePharma (Shanghai, China). Three different siRNAs were synthesized for each gene to ensure the efficiency of RNA interference, and their sequences are listed in Table 2. siATF6, siIRE1, sieIF2α, and the NC were transfected using X-tremeGENE siRNA transfection reagent (Roche, Sweden) according to the manufacturer’s instructions.

**TABLE 2:** siRNA sequences for IRE1, ATF6, eIF2α, and the NC

Target	siRNA	Sequence (5′–3′)
IRE1	1st	ACUACAUCCUGAAGAGAAATT
	2nd	GGACAGCUCUCAAGGGACATT
	3rd	GAGGGAGGAUCCAGAACUATT

ATF6	1st	GCACUUUGAUGCAGCACAUTT
	2nd	GCAAAGCAGCAGUCGAUUATT
	3rd	GCUGUCCAGUACACAGAAATT

eIF2α	1st	GCAGUCUCAGACCCAUCUATT
	2nd	CCAGAGGAAGCAAUCAAAUTT
	3rd	GCAGGUUUGAAUUGUUCUATT
	4th	GCCCAAAGUGGUCACAGAUTT

NC	Negative control	UUCUCCGAACGUGUCACGUTT

### RNA extraction and real-time quantitative RT-PCR.

Total RNA was isolated using a TransZol Up Plus RNA kit (catalog number ER501-01; Transgen, China). RNA was reverse transcribed using EasyScript one-step genomic DNA (gDNA) removal and cDNA synthesis supermix (catalog number AE311-01; Transgen, China). Quantitative PCR (qPCR) was performed in triplicate using 2× SYBR qPCR master mix (Bimake, Houston, TX, USA). The primers used for real-time quantitative RT-PCR (qRT-PCR) are listed in Table 3. All experimental data were obtained with a QuantStudio 3 real-time PCR system (Thermo Fisher Scientific, USA) and analyzed with QuantStudio Design and Analysis Software version 1.4.3 based on the ΔΔ*C_T_* method. Glyceraldehyde-3-phosphate dehydrogenase (GAPDH) was used as the internal control. To assess PHEV replication, standard-curve qPCR experiments were performed using the N gene of the PHEV genome as a standard. The following primers were designed based on the PHEV N gene used for quantification of the PHEV genome: 5′-TCTGGGAATCCTGACGAG-3′ (P1) and 5′-AGGCGCTGCAACACTTAC-3′ (P2).

**TABLE 3:** Real-time qRT-PCR primers used for UPR detection

Target	Primer	Sequence (5′–3′)
*Xbp1s*	P1	GAGTCCGCAGCAGGTG
	P2	GTGTCAGAGTCCATGGGA

*Xbp1u*	P1	TCCGCAGCACTCAGACTATGT
	P2	ATGCCCAAAAGGATATCAGACTC

*ERdj4*	P1	GGACAAAGAGGCAATGGG
	P2	CCTGGCGTGTGTGGAAGT

*p58* ^(^ *^IPK^* ^)^	P1	TGCCGATTACACTGCTGC
	P2	TCCTGGGCTCTCCTTCCT

*Atf4*	P1	CTGCCTTCTCCAGGTGGTTC
	P2	GGCTGCTGTCTTGTTTTGCT

*Gadd34*	P1	CCCCTCCAACTCTCCTTC
	P2	TGCCCTGTGTGCCTCTAC

*Grp78*	P1	CCTGCGTCGGTGTGTTCAAG
	P2	AAGGGTCATTCCAAGTGCG

*Grp94*	P1	AGTCGGGAAGCAACAGAGAA
	P2	TCTCCATGTTGCCAGACCAT

*Calreticulin*	P1	TGTTACCAAGGCTGCAGAGA
	P2	GGCCTCTACAGCTCATCCTT

*Chop*	P1	CCTCGCTCTCCAGATTCC
	P2	TCCTTCATGCGTTGCTTC

*Ire1*α	P1	TGCAGGCAGATCTGAAAAGG
	P2	TGAATGAAGCCAGCAGGAAG

*Atf6*	P1	TAGTTCTCAGTCCCCCCTTT
	P2	CCATGTTCTGTTTTGTTTCC

*Gapdh*	P1	CTCAACTACATGGTCTACATGTTC
	P2	ATTTGATGTTAGTGGGGTCTCGCTC

### Western blotting.

PHEV-infected or mock-infected mouse brains were collected, homogenized, and prepared as 10% (wt/vol) suspensions in PBS (pH 7.4). After centrifugation, the supernatants were collected and stored at −80°C. The brain extracts or cell samples were lysed with radioimmunoprecipitation assay (RIPA) lysis buffer (catalog number p0013B; Beyotime, China) with 1 mM phenylmethanesulfonyl fluoride (PMSF) (catalog number ST506; Beyotime, China) on ice for 30 min. The lysates were then subjected to 12% sodium dodecyl sulfate-polyacrylamide gel electrophoresis (SDS-PAGE). Separated proteins were transferred to polyvinylidene difluoride (PVDF) membranes, blocked with 5% skim milk, and then incubated with primary antibodies at 4°C overnight. After extensive washing with PBST (PBS, 0.1% Tween 20), the membranes were incubated with 1:5,000-diluted secondary antibodies for 1 h at 37°C. The membranes were detected using a Tanon 5200 automatic chemiluminescence imaging analysis system (Tanon, China). The intensity of each band was measured by ImageJ software (version 1.51j8).

### Immunofluorescence.

PHEV-infected or mock-infected monolayer N2a cells were collected at different times, fixed with 4% paraformaldehyde for 15 min, and permeabilized with a 0.2% Triton X-100 solution for 10 min at room temperature. The cells were blocked with 5% skim milk for 1 h at 37°C. The primary antibody was incubated at 4°C overnight, followed by the secondary antibody at 37°C for 1 h. Samples were mounted using antifade mounting medium with DAPI (4′,6-diamidino-2-phenylindole) (Beyotime, China) and observed under a confocal microscope.

### SUnSET assay.

A translation intensity measurement assay was conducted as previously described (50). Briefly, 10 μg/mL puromycin was added to the cell culture medium at the indicated time points, and the cells were incubated for another 10 min at 37°C with 5% CO_2_. The cells were then harvested and analyzed by Western blotting with the antipuromycin monoclonal antibody clone 12D10.

### Cell viability measurement.

Cells were seeded into 96-well plates and then treated with chemicals at the indicated concentrations for 24 h when cells were grown to 70 to 80% confluence. Cell viability was determined using cell counting kit 8 (CCK-8) (catalog number MA0218; Meilunbio) according to the manufacturer’s instructions.

### Statistical analysis.

All the results are expressed as the means ± standard deviations (SD), and statistical analysis was performed using two-tailed Student’s *t* test or one-way analysis of variance (ANOVA). A *P* value of <0.05 was considered statistically significant (*, *P* < 0.05; **, *P* < 0.01). Graphs were plotted and analyzed using GraphPad Prism v7.0 (GraphPad Software Inc., La Jolla, CA).
